# Edge-Preserving Denoising of Image Sequences

**DOI:** 10.3390/e23101332

**Published:** 2021-10-12

**Authors:** Fan Yi, Peihua Qiu

**Affiliations:** Department of Biostatistics, University of Florida, Gainesville, FL 32603, USA; pqiu@ufl.edu

**Keywords:** bandwidth selection, correlation, edge-preserving image denoising, image sequence, jump regression analysis, local smoothing, nonparametric regression, spatio-temporal data

## Abstract

To monitor the Earth’s surface, the satellite of the NASA Landsat program provides us image sequences of any region on the Earth constantly over time. These image sequences give us a unique resource to study the Earth’s surface, changes of the Earth resource over time, and their implications in agriculture, geology, forestry, and more. Besides natural sciences, image sequences are also commonly used in functional magnetic resonance imaging (fMRI) of medical studies for understanding the functioning of brains and other organs. In practice, observed images almost always contain noise and other contaminations. For a reliable subsequent image analysis, it is important to remove such contaminations in advance. This paper focuses on image sequence denoising, which has not been well-discussed in the literature yet. To this end, an edge-preserving image denoising procedure is suggested. The suggested method is based on a jump-preserving local smoothing procedure, in which the bandwidths are chosen such that the possible spatio-temporal correlations in the observed image intensities are accommodated properly. Both theoretical arguments and numerical studies show that this method works well in the various cases considered.

## 1. Introduction

**Figure 1:**
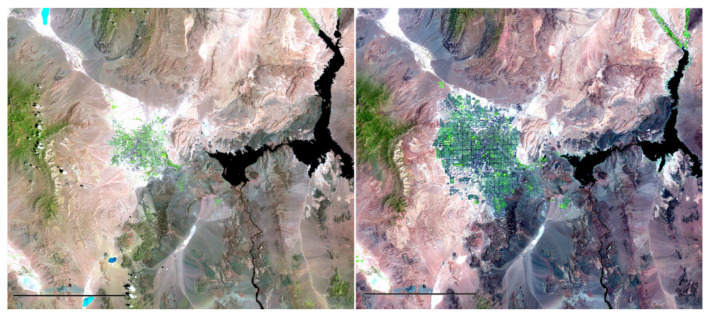
Two Landsat images of the Las Vegas area taken in 1984 (**left panel**) and 2007 (**right panel**).

The Landsat project, led by the US Geological Survey (USGS) and NASA, has launched eight satellites since 1972 to continuously provide scientifically valuable images of the Earth’s surface. These images can be freely accessed by researchers around the world (cf., Zanter [1]). This rich archive of Landsat images has become a major resource for scientific research about the Earth’s surface and its resources in different scientific disciplines, including forest science, climate science, agriculture, ecology, fire science, and many more. As an example, Figure 1 shows two images of the Las Vegas area in Nevada taken in 1984 and 2007, respectively. These two images clearly show the increasing urban sprawl of Las Vegas during the 23-year period, and consequently, the environment in that region has changed dramatically. The current satellite (i.e., the Landsat 8) can deliver an image of a given region roughly every 16 days. So, we have a sequence of images of that region collected sequentially over time, stored in the Landsat database, which is increasing all the time. Image sequences are commonly used in many other applications, including functional magnetic resonance imaging (fMRI) in neuroscience and quality control in manufacturing industries (Qiu [2]). In practice, observed images usually contain noise and other contaminations (Gonzalez and Woods [3]). For reliable subsequent image analyses, such contaminations should be removed in advance. In the image processing literature, the removal of noise from an observed image is referred to as image denoising. This paper focuses on image denoising for analyzing observed image sequences.

In the literature, there has been extensive discussion on image denoising (Qiu [4]). Many early methods in the computer science literature are based on the Markov random field (MRF) framework, in which observed image intensities of an image are assumed to have the Markov property that the observed intensity at a given pixel depends only on the observed intensities in a neighborhood of the given pixel (Geman and Geman [5]). Then, if the true image is assumed to have a prior distribution which is also an MRF, its posterior distribution would be an MRF too, and consequently, the true image can be estimated by the maximum a posteriori (MAP) estimator (e.g., Geman and Geman [5], Besag [6], Fessler et al. [7]). Other popular image denoising methods include those based on diffusion equations (e.g., Perona and Malik [8], Weickert [9]), total variation (Beck and Teboulle [10], Rudin et al. [11], Yuan et al. [12]), wavelet transformations (e.g., Chang et al. [13], Mrázek [14]), jump regression analysis (e.g., Gijbels et al. [15], Qiu [16], Qiu [17], Qiu and Mukherjee [18]), adaptive weights smoothing (e.g., Polzehl and Spokoiny [19]), spatial adaption (e.g., Kervrann and Boulanger [20]) and more. Besides noise removal, edge-preserving is important for image denoising because edges are important structures of the images. Some of the methods mentioned above can preserve edges well, such as the ones based on jump regression analysis, total variation, and wavelet transformations. Thorough surveys of popular edge-preserving image denoising methods can be found in Jain and Tyagi [21] and Qiu [4].

Although there are already some existing methods for edge-preserveing image denoising, almost all of them handle observed images taken at a single time point. So far, we have not found much discussion about denoising image sequences, which is the focus of the current paper. A given image sequence often describes a gradual change in appearance over time, subject to the underlying process. For instance, the sequence of images of the Las Vegas area acquired by the Landsat satellite (cf., Figure 1) describes the gradual change of the Earth’s surface in that area over time. As mentioned above, two consecutive images in the sequence acquired by the current Landsat satellite are only about 16 days apart. So, their difference should be very small. However, the images could be substantially different after a long period of time, as shown in Figure 1. In such applications, it should be reasonable to assume that edge locations in different images either do not change or change gradually over time. To handle such image sequences, the neighboring images should be useful when denoising the image at a given time point, or information in neighboring images should be shared during image denoising. By noticing such features of image sequences, we propose an edge-preserving image denoising procedure for analyzing image sequences in this paper. Our proposed method is based on the jump regression analysis (JRA) used for regression modeling when the underlying regression function has jumps or other singularities (Qiu [22]). It is a local smoothing procedure, and the possible spatio-temporal correlation in the observed image data has been accommodated properly in its construction. Both theoretical arguments and numerical studies show that this method works well in various different cases.

The remaining parts of the article are organized as follows. The proposed method is described in detail in Section 2. Its statistical properties and the numerical studies about its performance in different finite-sample cases are presented in Section 3. Several concluding remarks are provided in Section 4. Some technical details are given in Appendix A.

## 2. Materials and Methods

This section describes our proposed method in two parts. A JRA model for describing an image sequence and the model estimation are discussed in Section 2.1. Selection of several parameters used in model estimation is discussed in Section 2.2.

### 2.1. JRA Model and Its Estimation

To describe an image sequence, let us consider the following JRA model:

(1) $$Z_{ijk}=f\left(x_{i},y_{j};t_{k}\right)+\varepsilon_{ijk},\;i=1,2,\ldots ,n_{x},j=1,2,\ldots ,n_{y},k=1,2,\ldots ,n_{t},$$

where $Z_{ijk}$ is the observed image intensity level at the (i,j)-th pixel $\left(x_{i},y_{j}\right)$ and at the *k*-th time point $t_{k}$, $f(x_{i},y_{j};t_{k})$ is the true image intensity level, and $\varepsilon_{ijk}$ is the pointwise random noise with mean 0 and variance $\sigma^{2}$. In model (1), spatio-temporal data correlation is allowed, namely, $\left\{\varepsilon_{ijk}\right\}$ could be correlated over i,j and *k*. For image data, the pixel locations are usually regularly spaced. Without loss of generality, it is assumed that they are equally spaced in the design space Ω=[0,1]×[0,1], namely, $\left(x_{i},y_{j}\right)=\left(i/n_{x},j/n_{y}\right)$, for all *i* and *j*, where $n_{x}$ and $n_{y}$ are the numbers of rows and columns, respectively. The observation times $\left\{t_{k},k=1,2,\ldots ,n_{t}\right\}$ are also assumed to be equally spaced in the time interval [0,1]. The true image intensity function f(x,y;t), for (x,y)∈Ω, is continuous in the design space Ω at each t∈[0,1], except on the edges where it has jumps.

To estimate the unknown image intensity function f(x,y;t) in model (1), we consider using a local smoothing method, instead of a global smoothing method (e.g., smoothing spline method), because of a large amount of data involved in the current problem. Likewise, it has been well-discussed in the JRA literature that conventional smoothing methods (e.g., conventional local kernel smoothing methods) would not work well for estimating models like (1) where the true image intensity function f(x,y;t) has jumps at the edges, because the jumps would be blurred by such conventional methods (cf., Qiu [22]). In this paper, we suggest a jump-preserving local smoothing method for estimating (1), described in detail below. For a given point (x,y;t)∈Ω×[0,1], define a local neighborhood

$$\begin{array}{cc} O(x,y;t)= & \{\left(x^{{}^{\prime }},y^{{}^{\prime }};t^{{}^{\prime }}\right):\left(x^{{}^{\prime }},y^{{}^{\prime }};t^{{}^{\prime }}\right)\in \Omega \times \left[0,1\right],\\ & \sqrt{\frac{{\left(x^{{}^{\prime }}-x\right)}^{2}}{h_{x}^{2}}+\frac{{\left(y^{{}^{\prime }}-y\right)}^{2}}{h_{y}^{2}}}\leq 1,|t^{{}^{\prime }}-t\left|/h_{t}\leq 1\right\}, \end{array}$$

where $h_{x}$, $h_{y}$ and $h_{t}$ are the bandwidths in the x−, y−, and t−axis, respectively. In O(x,y;t), we first consider the following local linear kernel (LLK) smoothing procedure (Fan and Gijbels [23]):

(2) $$\begin{array}{cc} \min_{a,b,c,d}\sum_{i=1}^{n_{x}}\sum_{j=1}^{n_{y}}\sum_{k=1}^{n_{t}} & {\left\{Z_{ijk}-\left[a+b\left(x_{i}-x\right)+c\left(y_{j}-y\right)+d\left(t_{k}-t\right)\right]\right\}}^{2}\\ \; & K\left(\frac{x_{i}-x}{h_{x}},\frac{y_{j}-y}{h_{y}}\right)K\left(\frac{t_{k}-t}{h_{t}}\right), \end{array}$$

where K(v) is a density kernel function with the support {v:|v|≤1}. The solutions to (a,b,c,d) of the minimization problem (2) are denoted as $\hat{a}\left(x,y;t\right)$, $\hat{b}\left(x,y;t\right)$, $\hat{c}\left(x,y;t\right)$, and $\hat{d}\left(x,y;t\right)$, respectively. It can be checked that they have the following expressions:

(3) $$\left[\begin{array}{c} \hat{a}\left(x,y;t\right)\\ \hat{b}\left(x,y;t\right)\\ \hat{c}\left(x,y;t\right)\\ \hat{d}\left(x,y;t\right) \end{array}\right]={\left[\begin{array}{cccc} m_{000} & m_{100} & m_{010} & m_{001}\\ m_{100} & m_{200} & m_{110} & m_{101}\\ m_{010} & m_{110} & m_{020} & m_{011}\\ m_{001} & m_{101} & m_{011} & m_{002} \end{array}\right]}^{-1}\left[\begin{array}{c} \sum_{ijk}Z_{ijk}K_{ijk}\\ \sum_{ijk}\left(x_{i}-x\right)Z_{ijk}K_{ijk}\\ \sum_{ijk}\left(y_{j}-y\right)Z_{ijk}K_{ijk}\\ \sum_{ijk}\left(t_{k}-t\right)Z_{ijk}K_{ijk} \end{array}\right],$$

where $\sum_{ijk}$ denotes $\sum_{i=1}^{n_{x}}\sum_{j=1}^{n_{y}}\sum_{k=1}^{n_{t}}$, $K_{ijk}$ denotes $K\left(\frac{x_{i}-x}{h_{x}},\frac{y_{j}-y}{h_{y}}\right)K\left(\frac{t_{k}-t}{h_{t}}\right)$, and $m_{rsl}=\sum_{ijk}{\left(x_{i}-x\right)}^{r}{\left(y_{j}-y\right)}^{s}{\left(t_{k}-t\right)}^{l}K_{ijk}$, for r,s,l=0,1,2. The LLK estimator of f(x,y;t) is defined to be $\hat{a}\left(x,y;t\right)$. The estimated gradient direction of f(x,y;t) at (x,y;t) is $\hat{G}\left(x,y;t\right)={\left(\hat{b}\left(x,y;t\right),\hat{c}\left(x,y;t\right),\hat{d}\left(x,y;t\right)\right)}^{\prime }$ which indicates the direction in which the estimated plane in O(x,y;t) by the LLK procedure (2) increases the fastest. If there is an edge surface in O(x,y;t), then $\hat{G}\left(x,y;t\right)$ would be (approximately) orthogonal to that surface.

**Figure 2:**
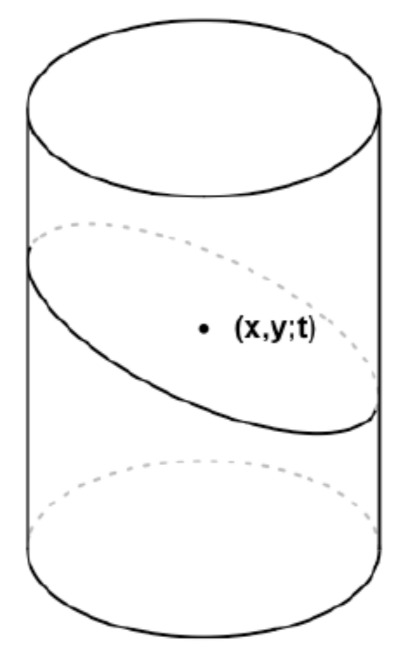
The neighborhood O(x,y;t) is divided into two parts by a plane that passes (x,y;t) and is perpendicular to the estimated gradient direction $\hat{G}\left(x,y;t\right)$.

In cases when there are no edges in the neighborhood O(x,y;t), $\hat{a}\left(x,y;t\right)$ would be a good estimate of f(x,y;t). Otherwise, it cannot be a good estimate because $\hat{a}\left(x,y;t\right)$ is a weighted average of all observed image intensities in O(x,y;t), the jumps in the image intensity surface would be smoothed out in the weighted average, and the estimate $\hat{a}\left(x,y;t\right)$ would be biased for estimating f(x,y;t). To overcome that limitation, we consider the following one-sided smoothing idea. Let O(x,y;t) be divided into two parts $O^{\left(1\right)}\left(x,y;t\right)$ and $O^{\left(2\right)}\left(x,y;t\right)$ by a plane that passes (x,y;t) and is perpendicular to $\hat{G}\left(x,y;t\right)$. See Figure 2 for an example.

Then, in cases when there is an edge surface in O(x,y;t), that plane would be (approximately) parallel to the edge surface. Consequently, at least one of $O^{\left(1\right)}\left(x,y;t\right)$ and $O^{\left(2\right)}\left(x,y;t\right)$ would be (mostly) located on a single side of the edge surface in such cases. Now, let us consider the following one-sided LLK smoothing procedure: for l=1,2,

(4) $$\begin{array}{cc} \min_{a,b,c,d}\sum_{\left(x_{i},y_{j};t_{k}\right)\in O^{\left(l\right)}\left(x,y;t\right)}\; & {\left\{Z_{ijk}-\left[a+b\left(x_{i}-x\right)+c\left(y_{j}-y\right)+d\left(t_{k}-t\right)\right]\right\}}^{2}\\ \; & K\left(\frac{x_{i}-x}{h_{x}},\frac{y_{j}-y}{h_{y}}\right)K\left(\frac{t_{k}-t}{h_{t}}\right). \end{array}$$

The solutions of (4) to (a,b,c,d) are denoted as $({\hat{a}}^{\left(l\right)}\left(x,y;t\right),$${\hat{b}}^{\left(l\right)}\left(x,y;t\right),$${\hat{c}}^{\left(l\right)}\left(x,y;t\right),$${\hat{d}}^{\left(l\right)}\left(x,y;t\right))$, for l=1,2. Intuitively, when there are no edges in O(x,y;t), $\hat{a}\left(x,y;t\right)$, ${\hat{a}}^{\left(1\right)}\left(x,y;t\right)$ and ${\hat{a}}^{\left(2\right)}\left(x,y;t\right)$ are all consistent estimates of f(x,y;t) under some regular conditions. In such cases, $\hat{a}\left(x,y;t\right)$ would be preferred since it averages more observations and consequently it would have a smaller variance. When there are edges in O(x,y;t), $\hat{a}\left(x,y;t\right)$ would not be a good estimate of f(x,y;t) as explained above, but one of ${\hat{a}}^{\left(1\right)}\left(x,y;t\right)$ and ${\hat{a}}^{\left(2\right)}\left(x,y;t\right)$ should estimate f(x,y;t) well. Therefore, in all cases, at least one of the three estimators $\hat{a}\left(x,y;t\right)$, ${\hat{a}}^{\left(1\right)}\left(x,y;t\right)$ and ${\hat{a}}^{\left(2\right)}\left(x,y;t\right)$ should estimate f(x,y;t) well.

Next, we need to choose a good estimator from $\hat{a}\left(x,y;t\right)$, ${\hat{a}}^{\left(1\right)}\left(x,y;t\right)$ and ${\hat{a}}^{\left(2\right)}\left(x,y;t\right)$ based on the observed data, which is not straightforward, partly because we do not know in advance whether there are edges in the neighborhood O(x,y;t) and whether the edges are mostly contained in $O^{\left(1\right)}\left(x,y;t\right)$ or $O^{\left(2\right)}\left(x,y;t\right)$ if the answer to the first question is positive. To overcome this difficulty, let us consider the following weighted residual mean squares (WRMS) of the fitted local plane by the LLK procedure (2):

(5) $$\begin{array}{cc} e(x,y;t)= & \{\sum_{ijk}[Z_{ijk}-\hat{a}\left(x,y;t\right)-\hat{b}\left(x,y;t\right)\left(x_{i}-x\right)-\hat{c}\left(x,y;t\right)\left(y_{j}-y\right)-\\ \; & \hat{d}\left(x,y;t\right)\left(t_{k}-t\right){]}^{2}K_{ijk}\}/\sum_{ijk}K_{ijk}. \end{array}$$

The above WRMS measures how well the fitted local plane describes the observed data in O(x,y;t). If there are edges in O(x,y;t), this quantity would be relatively large, due mainly to the jumps in the image intensity surface. Otherwise, it would be relatively small. So, the quantity e(x,y;t) contains useful information about the existence of edges in O(x,y;t). Similarly, we can define WRMS values for the two one-sided local planes fitted in $O^{\left(1\right)}\left(x,y;t\right)$ and $O^{\left(2\right)}\left(x,y;t\right)$. They are denoted as $e^{\left(1\right)}\left(x,y;t\right)$ and $e^{\left(2\right)}\left(x,y;t\right)$. Based on these WRMS values, we define our edge-preserving estimator of f(x,y;t) to be

(6) $$\begin{array}{cc} \hat{f}\left(x,y;t\right) & =\hat{a}\left(x,y;t\right)I\left(D\left(x,y;t\right)\leq u\right)\\ \; & +{\hat{a}}^{\left(1\right)}\left(x,y;t\right)I\left(D\left(x,y;t\right)>u\right)I\left(e^{\left(1\right)}\left(x,y;t\right)<e^{\left(2\right)}\left(x,y;t\right)\right)\\ \; & +{\hat{a}}^{\left(2\right)}\left(x,y;t\right)I\left(D\left(x,y;t\right)>u\right)I\left(e^{\left(1\right)}\left(x,y;t\right)>e^{\left(2\right)}\left(x,y;t\right)\right)\\ \; & +\frac{{\hat{a}}^{\left(1\right)}\left(x,y;t\right)+{\hat{a}}^{\left(2\right)}\left(x,y;t\right)}{2}I\left(D\left(x,y;t\right)>u\right)I\left(e^{\left(1\right)}\left(x,y;t\right)=e^{\left(2\right)}\left(x,y;t\right)\right), \end{array}$$

where $D\left(x,y;t\right)=\max(e\left(x,y;t\right)-e^{\left(1\right)}\left(x,y;t\right),e\left(x,y;t\right)-e^{\left(2\right)}\left(x,y;t\right))$, I(·) is the indicator function, and u>0 is a threshold parameter. By (6), it is obvious that $\hat{f}\left(x,y;t\right)$ is defined to be one of $\hat{a}\left(x,y;t\right)$, ${\hat{a}}^{\left(1\right)}\left(x,y;t\right)$ and ${\hat{a}}^{\left(2\right)}\left(x,y;t\right)$. The quantity $\hat{a}\left(x,y;t\right)$, which is obtained from the entire neighborhood O(x,y;t), is chosen if the observed data indicate no edges in O(x,y;t), supported by the event D(x,y;t)≤u. Otherwise, one of the two one-sided quantities, ${\hat{a}}^{\left(1\right)}\left(x,y;t\right)$ and ${\hat{a}}^{\left(2\right)}\left(x,y;t\right)$, with a smaller WRMS value is chosen. Although, theoretically, the event $\left(e^{\left(1\right)}\left(x,y;t\right)=e^{\left(2\right)}\left(x,y;t\right)\right)$ would have probability zero of happening, the last term on the right-hand-side of (6) is still included for completeness of the definition of $\hat{f}\left(x,y;t\right)$ and for the consideration that $e^{\left(1\right)}\left(x,y;t\right)$ and $e^{\left(2\right)}\left(x,y;t\right)$ could be considered the same in certain algorithms when their values are close.

### 2.2. Parameter Selection

In our proposed method described in Section 2.1, there are four parameters; $h_{x}$, $h_{y}$, $h_{t}$ and *u*, that need to be chosen properly in advance. For that purpose, it is natural to consider the cross validation (CV) procedure, especially in the current research problem where the observed data are quite large in size. However, it has been well-demonstrated in the literature that the conventional CV procedure would not work well in cases when the observed data are autocorrelated, because it cannot effectively distinguish the data correlation structure from the mean structure (cf., Altman [24], Opsomer et al. [25]). In the current problem, spatio-temperal data correlation is possible in almost all applications. Thus, the conventional CV procedure is not feasible in such cases. In the univariate regression setup, Brabanter et al. [26] suggested a modified CV procedure for choosing smoothing parameters in cases with correlated data. This procedure is generalized here for choosing the parameters $h_{x}$, $h_{y}$, $h_{t}$ and *u* used in the proposed method, which is described below. Let the modified CV score for choosing $h_{x}$, $h_{y}$, $h_{t}$ and *u* be defined as

(7) $$CV\left(h_{x},h_{y},h_{t},u\right)=\frac{1}{n_{x}n_{y}n_{t}}\sum_{ijk}{\left[{\hat{f}}_{-(ijk)}\left(x_{i},y_{j};t_{k}\right)-Z\left(x_{i},y_{j};t_{k}\right)\right]}^{2},$$

where ${\hat{f}}_{-(ijk)}\left(x_{i},y_{j};t_{k}\right)$ is the leave-one-out estimate of $f(x_{i},y_{j};t_{k})$ by (2)–(6) after the observation $Z_{ijk}$ is removed from the estimation process and after the kernel function is replaced by the so-called ϵ-optimal bimodal kernel function $K_{\epsilon }\left(v\right)$ defined to be

(8) $$K_{\epsilon }\left(v\right)=\frac{4}{4-3\epsilon -\epsilon^{3}}\times \left\{\begin{array}{cc} \frac{3}{4}\left(1-v^{2}\right)I\left(|v|\leq 1\right), & if|v|\geq \epsilon ,\\ \frac{3(1-\epsilon^{2})}{4\epsilon }\left|v\right|, & if|v|<\epsilon , \end{array}\right.$$

where 0<ϵ<1 is a parameter. Based on a large simulation study, Brabanter et al. [26] suggested choosing ϵ to be 0.1, which is adopted in this paper. Then, by the above modified CV procedure, (7) and (8), the parameters $h_{x}$, $h_{y}$, $h_{t}$ and *u* can be chosen by minimizing the modified CV score $CV(h_{x},h_{y},h_{t},u)$.

## 3. Results

### 3.1. Statistical Properties

In this part, we discuss some statistical properties of the proposed edge-preserving image sequence denoising method (2)–(6). First, we have the following proposition.

**Proposition** **1.** *Assume that i) the kernel function K(v) used in (2) is a Lipschitz-1 continuous density function, and ii) the noise terms $\left\{\varepsilon_{ijk},i=1,2,\ldots ,n_{x},j=1,2,\ldots ,n_{y},k=1,2,\ldots ,n_{t}\right\}$ in model (1) form a strong mixing stochastic process with the following strong mixing coefficients:*

$$\begin{array}{ccc} \alpha (d) & = & \underset{\left(ijk\right),\left(i^{{}^{\prime }}j^{{}^{\prime }}k^{{}^{\prime }}\right)}{\sup}\underset{A,B}{\sup}\{\left|P\left(A\cap B\right)-P\left(A\right)P\left(B\right)\right|,A\in \sigma \left(\varepsilon_{ijk}\right),B\in \sigma \left(\varepsilon_{i^{{}^{\prime }}j^{{}^{\prime }}k^{{}^{\prime }}}\right),\\ & & \max\{|i-i^{{}^{\prime }}|,|j-j^{{}^{\prime }}|,|k-k^{{}^{\prime }}|\}>d\}, \end{array}$$

*which have the property that $\alpha \left(d\right)\leq c_{1}\sigma^{2}\rho^{c_{2}d}$, where $c_{1},c_{2}>0$ and 0<ρ<1 are constants, and iii) $E(\varepsilon_{111}^{6})<\infty$. Let $N=n_{x}n_{y}n_{t}$, $H=h_{x}h_{y}h_{t}$, $n_{min}=\min\left(n_{x},n_{y},n_{t}\right)$, and $h_{min}=\min\left(h_{x},h_{y},h_{t}\right)$. Then, for any $\left(x,y;t\right)\in \Omega_{h}=\left[h_{x},1-h_{x}\right]\times \left[h_{y},1-h_{y}\right]\times \left[h_{t},1-h_{t}\right]$, we have*

$$|\frac{1}{NH}\sum_{ijk}K\left(\frac{x_{i}-x}{h_{x}},\frac{y_{i}-y}{h_{y}}\right)K\left(\frac{t_{i}-t}{h_{t}}\right)-1|=O\left(\frac{1}{n_{min}h_{min}}\right),$$

$$E\left[|\frac{1}{NH}\sum_{ijk}\varepsilon_{ijk}K\left(\frac{x_{i}-x}{h_{x}},\frac{y_{i}-y}{h_{y}}\right)K\left(\frac{t_{i}-t}{h_{t}}\right){|}^{2}\right]=O\left(\frac{1}{NH}\right),$$

$$E\left[|\frac{1}{NH}\sum_{ijk}\left(\varepsilon_{ijk}^{2}-\sigma^{2}\right)K\left(\frac{x_{i}-x}{h_{x}},\frac{y_{i}-y}{h_{y}}\right)K\left(\frac{t_{i}-t}{h_{t}}\right){|}^{2}\right]=O\left(\frac{1}{NH}\right).$$

Based on the results in Proposition 1, we can derive the following properties of the LLK estimates defined in (3).

**Theorem** **1.** *Besides the conditions in Proposition 1, we further assume that the true image intensity function f(x,y;t) has continuous first-order partial derivatives with respect to x, y and t in the design space* Ω *except at the edge curves. Then, for any $\left(x,y;t\right)\in \Omega_{h}\setminus J_{h}$, we have*

$$\left[\begin{array}{c} \hat{a}\left(x,y;t\right)\\ \hat{b}\left(x,y;t\right)\\ \hat{c}\left(x,y;t\right)\\ \hat{d}\left(x,y;t\right) \end{array}\right]=\left[\begin{array}{c} f(x,y;t)\\ f_{x}^{{}^{\prime }}\left(x,y;t\right)\\ f_{y}^{{}^{\prime }}\left(x,y;t\right)\\ f_{t}^{{}^{\prime }}\left(x,y;t\right) \end{array}\right]+\left[\begin{array}{c} O(h_{x}^{2}+h_{y}^{2}+h_{t}^{2})\\ O(\frac{h_{x}^{2}+h_{y}^{2}+h_{t}^{2}}{h_{x}})\\ O(\frac{h_{x}^{2}+h_{y}^{2}+h_{t}^{2}}{h_{y}})\\ O(\frac{h_{x}^{2}+h_{y}^{2}+h_{t}^{2}}{h_{t}}) \end{array}\right]+\left[\begin{array}{c} O_{p}\left(\frac{1}{\sqrt{NH}}\right)\\ O_{p}\left(\frac{1}{h_{x}\sqrt{NH}}\right)\\ O_{p}\left(\frac{1}{h_{y}\sqrt{NH}}\right)\\ O_{p}\left(\frac{1}{h_{t}\sqrt{NH}}\right) \end{array}\right].$$

*for any $\left(x,y,t\right)\in J_{h}\setminus S_{h}$, we have*

(9) $$\begin{array}{cc} \left[\begin{array}{c} \hat{a}\left(x,y;t\right)\\ \hat{b}\left(x,y;t\right)\\ \hat{c}\left(x,y;t\right)\\ \hat{d}\left(x,y;t\right) \end{array}\right]= & \left[\begin{array}{c} f_{-}\left(x_{\tau },y_{\tau };t_{\tau }\right)+d_{\tau }\xi_{000}^{\left(2\right)}\\ \frac{d_{\tau }}{\xi_{200}h_{x}}\xi_{100}^{\left(2\right)}\\ \frac{d_{\tau }}{\xi_{020}h_{y}}\xi_{010}^{\left(2\right)}\\ \frac{d_{\tau }}{\xi_{002}h_{t}}\xi_{001}^{\left(2\right)} \end{array}\right]+\left[\begin{array}{c} O(\sqrt{h_{x}^{2}+h_{y}^{2}+h_{t}^{2}})\\ O(\frac{\sqrt{h_{x}^{2}+h_{y}^{2}+h_{t}^{2}}}{h_{x}})\\ O(\frac{\sqrt{h_{x}^{2}+h_{y}^{2}+h_{t}^{2}}}{h_{y}})\\ O(\frac{\sqrt{h_{x}^{2}+h_{y}^{2}+h_{t}^{2}}}{h_{t}}) \end{array}\right]+\left[\begin{array}{c} O_{p}\left(\frac{1}{\sqrt{NH}}\right)\\ O_{p}\left(\frac{1}{h_{x}\sqrt{NH}}\right)\\ O_{p}\left(\frac{1}{h_{y}\sqrt{NH}}\right)\\ O_{p}\left(\frac{1}{h_{t}\sqrt{NH}}\right) \end{array}\right], \end{array}$$

where $\xi_{rsl}=\int_{\Omega \times [0,1]}u^{r}v^{s}w^{l}K\left(u,v\right)K\left(w\right)\;dudvdw$, $\xi_{rsl}^{\left(2\right)}=\int_{Q^{\left(2\right)}}u^{r}v^{s}w^{l}K\left(u,v\right)K\left(w\right)\;dudvdw$, for r,s,l=0,1,2, J is the closure of the set of all jump points of f(x,y;t), $J_{h}=\{\left(x,y;t\right):\left(x,y;t\right)\in \Omega_{h},\sqrt{{\left(x-x^{*}\right)}^{2}/h_{x}^{2}+{\left(y-y^{*}\right)}^{2}/h_{y}^{2}}\leq 1,|t-t^{*}\left|/h_{t}\leq 1,forany\left(x^{*},y^{*},t^{*}\right)\in J\right\}$, S is the set of singular points in J, including the crossing points of two or more edges, points on an edge surface at which the edge surface does not have a unique tangent surface, and points in J at which the jump sizes in f(x,y;t) are zero, $S_{h}=\{\left(x,y;t\right):\left(x,y;t\right)\in \Omega_{h}$, $\sqrt{{\left(x-x^{*}\right)}^{2}/h_{x}^{2}+{\left(y-y^{*}\right)}^{2}/h_{y}^{2}}\leq 1,|t-t^{*}\left|/h_{t}\leq 1,for\;any\;\left(x^{*},y^{*},t^{*}\right)\in S\right\}$, $(x_{\tau },y_{\tau };t_{\tau })\in J\setminus S$ is the projection of (x,y;t) to J with the Euclidean distance between the two points being $c\sqrt{h_{x}^{2}+h_{y}^{2}+h_{t}^{2}}$, for a constant 0<c<1, and $f_{-}\left(x_{\tau },y_{\tau };t_{\tau }\right)$ is the smaller one of the two one-sided limits of f(x,y;t) at $\left(x_{\tau },y_{\tau };t_{\tau }\right)$. In cases when O(x,y;t) contains jumps, without loss of generality, it is assumed that O(x,y;t) is divided by the edge surface into two parts $I_{1}$ and $I_{2}$ with a positive jump size $d_{\tau }$ from $I_{1}$ to $I_{2}$ at $\left(x_{\tau },y_{\tau };t_{\tau }\right)$, and $Q^{\left(1\right)}$ and $Q^{\left(2\right)}$ are the two corresponding parts in the support of K(u,v)K(w).

The next two theorems establish the consistency of the proposed edge-preserving image denoising procedure (2)–(6). First, we have the following theorem about the WRMS values defined in (5).

**Theorem** **2.** *Assume that the conditions in Theorem 1 are satisfied, $h_{x}^{2}+h_{y}^{2}+h_{t}^{2}=o\left(1\right)$, $\left(h_{x}^{2}+h_{y}^{2}+h_{t}^{2}\right)/h_{min}=o\left(1\right)$, 1/(NH)=o(1) and $1/\left(NHh_{min}^{2}\right)=o\left(1\right)$. Then, we have the following results: for any $\left(x,y;t\right)\in \Omega_{h}\setminus J_{h}$,*

(10) $$\begin{array}{cc} e(x,y;t) & =\sigma^{2}+o_{p}\left(1\right),\\ e^{\left(l\right)}\left(x,y;t\right) & =\sigma^{2}+o_{p}\left(1\right),\;forl=1,2; \end{array}$$

*for any $\left(x,y;t\right)\in J_{h}\setminus S_{h}$,*

(11) $$\begin{array}{cc} e(x,y;t) & =\sigma^{2}+d_{\tau }C_{\tau }^{2}+o_{p}\left(1\right),\\ e^{\left(l\right)}\left(x,y;t\right) & =\sigma^{2}+d_{\tau }{\left[C_{\tau }^{\left(l\right)}\right]}^{2}+o_{p}\left(1\right),\;forl=1,2, \end{array}$$

*where*

$$\begin{array}{ccc} C_{\tau } & = & (\int \int \int_{Q^{\left(1\right)}}{\left[\xi_{000}^{\left(2\right)}+\frac{\xi_{100}^{\left(2\right)}}{\xi_{200}}u+\frac{\xi_{010}^{\left(2\right)}}{\xi_{020}}v+\frac{\xi_{001}^{\left(2\right)}}{\xi_{002}}w\right]}^{2}K\left(u,v\right)K\left(w\right)dudvdw+\\ & & \int \int \int_{Q^{\left(2\right)}}{\left[1-\xi_{000}^{\left(2\right)}-\frac{\xi_{100}^{\left(2\right)}}{\xi_{200}}u-\frac{\xi_{010}^{\left(2\right)}}{\xi_{020}}v-\frac{\xi_{001}^{\left(2\right)}}{\xi_{002}}w\right]}^{2}K\left(u,v\right)K\left(w\right)dudvdw{)}^{1/2}. \end{array}$$

*and*

$$\begin{array}{ccc} C_{\tau }^{\left(l\right)} & = & (2\int \int \int_{Q^{\left(1l\right)}}{\left[B_{0l}+\frac{B_{1l}}{\xi_{200}}u+\frac{B_{2l}}{\xi_{020}}v+\frac{B_{3l}}{\xi_{002}}w\right]}^{2}K\left(u,v\right)K\left(w\right)dudvdw+\\ & & 2\int \int \int_{Q^{\left(2l\right)}}{\left[1-B_{0l}-\frac{B_{1l}}{\xi_{200}}u-\frac{B_{2l}}{\xi_{020}}v-\frac{B_{3l}}{\xi_{002}}w\right]}^{2}K\left(u,v\right)K\left(w\right)dudvdw{)}^{1/2}. \end{array}$$

*with the quantities $Q^{\left(1l\right)}$, $Q^{\left(2l\right)}$, $B_{0l}$, $B_{1l}$, $B_{2l}$ and $B_{3l}$ defined as follows. Let $\vec{g}=(\frac{d_{\tau }}{\xi_{200}h_{x}}\xi_{100}^{\left(2\right)}$, $\frac{d_{\tau }}{\xi_{020}h_{y}}\xi_{010}^{\left(2\right)}$, $\frac{d_{\tau }}{\xi_{002}h_{t}}\xi_{001}^{\left(2\right)})$. Then, from (9), $\vec{g}$ is actually the asymptotic direction of the gradient vector $\hat{G}\left(x,y;t\right)$. Let ${\tilde{O}}^{\left(l\right)}\left(x,y;t\right)$, for l=1,2, be two halves of the neighborhood O(x,y;t) separated by a plane passing the point (x,y;t) in the direction perpendicular to $\vec{g}$ and ${\tilde{Q}}^{\left(l\right)}$ be the two corresponding parts in the support of K(u,v)K(w). Then, $Q^{\left(1l\right)}=Q^{\left(1\right)}\cap {\tilde{Q}}^{\left(l\right)}$, $Q^{\left(2l\right)}=Q^{\left(2\right)}\cap {\tilde{Q}}^{\left(l\right)}$, $B_{0l}=\int \int \int_{Q^{\left(2l\right)}}K\left(u,v\right)K\left(w\right)dudvdw$, $B_{1l}=\int \int \int_{Q^{\left(2l\right)}}uK\left(u,v\right)K\left(w\right)dudvdw$, $B_{2l}=\int \int \int_{Q^{\left(2l\right)}}vK\left(u,v\right)K\left(w\right)dudvdw$, and $B_{3l}=\int \int \int_{Q^{\left(2l\right)}}wK\left(u,v\right)K\left(w\right)dudvdw$, for l=1,2.*

**Theorem** **3.** *Under the conditions in Theorem 2 and the extra assumption that threshold parameter $u=u_{N}\to 0$ as N→∞, we have, for any $\left(x,y;t\right)\in \Omega_{h}$,*

$$\hat{f}\left(x,y;t\right)=f\left(x,y;t\right)+o_{p}\left(1\right).$$

The proofs of these theoretical results are given in Appendix A.

### 3.2. Numerical Studies

**Figure 3:**
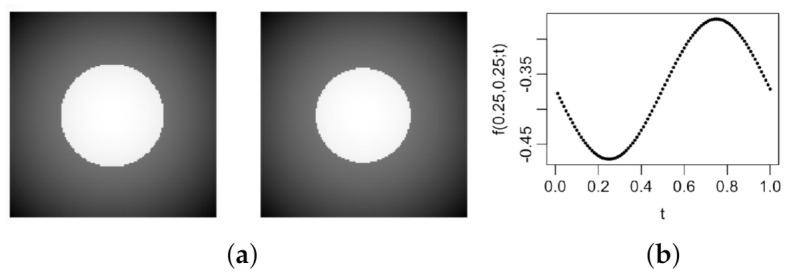
(**a**) The true image intensity function f(x,y;t) at t=0.01 (left) and t=0.25 (right). (**b**) The temporal profile f(0.25,0.25;t) when *t* changes in [0,1].

In this part, we study the numerical performance of our proposed method for denoising an image sequence. First, we consider a simulation example in which the true image intensity function in model (1) has the following expression:

$$f\left(x,y;t\right)=\left\{\begin{array}{cc} -2{\left(x-0.5\right)}^{2}-2{\left(y-0.5\right)}^{2}-0.1\sin\left(2\pi t\right)+1, & ifr\left(x,y;t\right)\leq 0.25^{2},\\ -2{\left(x-0.5\right)}^{2}-2{\left(y-0.5\right)}^{2}-0.1\sin\left(2\pi t\right), & otherwise, \end{array}\right.$$

where $r\left(x,y;t\right)={\left(x-0.5\right)}^{2}+{\left(y-0.5\right)}^{2}+0.01\sin\left(2\pi t\right)$, (x,y)∈Ω=[0,1]×[0,1], and t∈[0,1]. At a given value of *t*, f(x,y;t) has a circular edge curve $r\left(x,y;t\right)=0.25^{2}$ with a constant jump size 1 in f(x,y;t) at the edges. The radius of the circular edge curve, $\sqrt{0.25^{2}-0.01\sin\left(2\pi t\right)}$, changes periodically over t∈[0,1]. The image intensity function f(x,y;t) at t=0.01 and 0.25 and its temporal profile f(0.25,0.25;t) are shown in Figure 3. It can be seen that both the image intensity level at a given pixel and the edge curve change gradually when *t* changes in [0,1].

In model (1), the random errors $\left\{\varepsilon_{ijk},i=1,2,\ldots ,n_{x},j=1,2,\ldots ,n_{y},k=1,2,\ldots ,n_{t}\right\}$ are generated by the function spatialnoise() in the R-package neuRosim (cf., Welvaert et al. [27]). In that R function, there are two parameters ρ and σ to specify in advance, where ρ controls the data autocorrelation in all three dimensions and σ is the common standard deviation of the random errors. In all our examples, σ is fixed at 0.1, 0.2 or 0.3, and ρ is fixed at 0.1, 0.3 or 0.5, to study the possible impact of data noise level and data correlation on the performance of the proposed method. Without loss of generality, we set $n_{x}=n_{y}$ in all examples. In the model estimation procedure (2)–(6), we set $h_{x}=h_{y}$, and the kernel function K(v) is chosen to be the following truncated Gaussian density function:

$$K\left(v\right)=\left\{\begin{array}{cc} \frac{\exp\left(-v^{2}/2\right)-\exp\left(-0.5\right)}{2\pi -3\pi \exp(-0.5)}, & if|v|\leq 1,\\ 0, & otherwise. \end{array}\right.$$

In cases when σ=0.1, 0.2 or 0.3, $n_{x}=64$ or 128, $n_{t}=50$ or 100, ρ=0.1, 0.3 or 0.5, the MSE values of the estimator $\hat{f}\left(x,y;t\right)$ defined in (6) are presented in Table 1, along with the corresponding parameters $h_{x}$, $h_{t}$ and *u* selected by the modified CV procedure (7) and (8). In each case considered, the MSE value is computed based on 10 replicated simulations. For comparison purposes, the optimal MSE value of the estimator $\hat{f}\left(x,y;t\right)$, when its parameters ($h_{x}$, $h_{t}$ and *u*) are chosen such that the MSE value reaches the minimum in each case considered, is also presented in the table, along with the corresponding parameter values. From the table, we can draw the following conclusions. (i) The MSE values are smaller when either $n_{x}$ or $n_{t}$ is larger, which confirms the consistency results discussed in Section 3.1. (ii) When ρ is larger (i.e., the spatio-temporal data correlation is stronger), the MSE values are larger. So, data correlation does have an impact on the performance of the proposed method, which is intuitively reasonable. (iii) By comparing the MSE and the optimal MSE values, we can see that the MSE values are usually larger than their optimal values, but their differences are not that big in almost all cases considered. This conclusion indicates that the modified CV procedure (7) and (8) for determining the values of the parameters $\left(h_{x},h_{t},u\right)$ is quite effective. (iv) The parameter values chosen by the modified CV procedure (7) and (8) are quite close to the optimal parameter values in most cases considered.

Next, we compare our proposed method, denoted as NEW, with some alternative methods described below. The first alternative method is the conventional LLK procedure (2), by which f(x,y;t) is estimated by $\hat{a}\left(x,y;t\right)$ defined in (3). Its bandwidths are chosen by the conventional CV procedure, without considering any possible spatio-temporal data correlation. As explained in Section 2.1, this estimator would blur edges while removing noise. The second alternative method is to use $\hat{a}\left(x,y;t\right)$ for estimating f(x,y;t), but its bandwidths are chosen by the modified CV procedure (7) and (8). The above two alternative methods are denoted as LLK-C and LLK, respectively, where LLK-C denotes the first conventional LLK procedure that does not accommodate data correlation. The third alternative method is the one by Gijbels et al. [15] which is used for edge-preserving image denoising of a single image. To apply this method to the current problem, individual images collected at different time points can be denoised by it separately. This method assumes that the observed image intensities at different pixels are independent of each other, and thus their bandwidths can be chosen by the conventional CV procedure. This method is denoted as GLQ. The fourth alternative method is to use $\hat{f}\left(x,y;t\right)$ in (6) to estimate f(x,y;t), but the parameters $\left(h_{x},h_{t},u\right)$ are chosen by the conventional CV procedure. This method is denoted as NEW-C. By considering all these four alternative methods (i.e., LLK-C, LLK, GLQ and NEW-C), we can check whether the current problem to denoise an image sequence can be handled properly by the conventional LLK procedure with or without using the modified CV procedure, by an existing edge-preserving image denoising method designed for denoising a single image, or by the proposed method without considering the possible spatio-temporal data correlation. To evaluate their performance, in addition to the regular MSE criterion, we also consider the following edge-preservation (EP) criterion originally discussed in Hall and Qiu [28]:

$$EP\left(\hat{f}\right)=\left|JS\left(\hat{f}\right)-JS\left(f\right)\right|/JS\left(f\right),$$

where

$$\begin{array}{cc} JS(f)= & \frac{1}{\left(n_{x}-2\right)\left(n_{y}-2\right)\left(n_{t}-2\right)}\sum_{i=2}^{n_{x}-1}\sum_{j=2}^{n_{y}-1}\sum_{k=2}^{n_{t}-1}({\left[f\left(x_{i+1},y_{j};t_{k}\right)-f\left(x_{i-1},y_{j};t_{k}\right)\right]}^{2}+\\ \; & {\left[f\left(x_{i},y_{j+1};t_{k}\right)-f\left(x_{i},y_{j-1};t_{k}\right)\right]}^{2}+{\left[f\left(x_{i},y_{j};t_{k+1}\right)-f\left(x_{i},y_{j};t_{k-1}\right)\right]}^{2}{)}^{1/2}, \end{array}$$

and JS($\hat{f}$) is defined similarly. According to Hall and Qiu [28], JS(f) is a reasonable measure of the cumulative jump magnitude of *f* at the edge locations. So, $EP(\hat{f})$ provides a measure of the percentage of the cumulative jump magnitude of *f* that has been lost during data smoothing by using the estimator $\hat{f}$. By this explanation, the smaller its value, the better. In cases when σ=0.1, 0.2 or 0.3, $n_{x}=128$, $n_{t}=100$, and ρ=0.1, 0.3 or 0.5, the MSE and EP values of the related methods are presented in Table 2. From the table, it can be seen that the proposed method NEW has the smallest MSE values with quite large margins among all five methods in all cases considered, except the case when σ=0.1 and ρ=0.1 where NEW-C has a lightly smaller MSE value than that of NEW due to the weak data correlation in that case. Likewise, NEW has much smaller EP values in all cases considered, compared to the four competing methods. This example confirms that it is necessary to consider edge-preserving procedures when denoising image sequences and the possible spatio-temporal data correlation should be taken into account during the denoising process. It also confirms the benefit to share useful information among neighboring images when denoising an image sequence.

**Figure 4:**
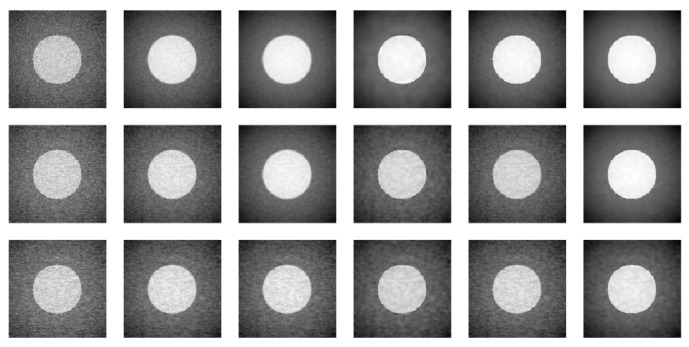
The first column shows the observed images at t=0.5 when σ=0.2 and ρ=0.1 (1st row), 0.3 (2nd row), and 0.5 (3rd row). Second to sixth columns show the denoised images by LLK-C, LLK, GLQ, NEW-C and NEW, respectively.

In the cases when σ=0.2 and ρ=0.1, 0.3 or 0.5, Figure 4 shows the observed images at t=0.5 in the first column, and the denoised images by the methods LLK-C, LLK, GLQ, NEW-C and NEW in columns 2–6. From the figure, it can be seen that the denoised images by NEW are the best in removing noise and preserving edges. As a comparison, the denoised images by LLK-C, and NEW-C are quite noisy because their selected bandwidths by the conventional CV procedure are relatively small due to the fact the conventional CV procedure cannot distinguish the data correlation from the mean structure, as discussed in Section 2.2. The denoised images by LLK are quite blurry because the method does not take the edges into account when denoising the images. The denoised images by GLQ are quite blurry as well since GLQ denoises individual images at different time points separately and the serial data correlation is ignored in this method.

**Figure 5:**
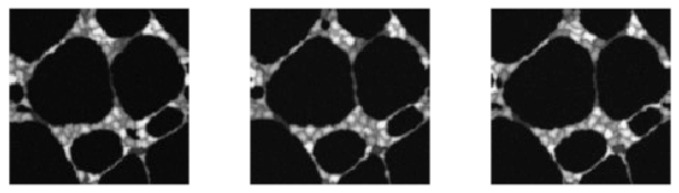
The 1st, 50th and 100th cell images of the image sequence for describing a vasculogenesis process.

Next, we apply the proposed method NEW and the four alternative methods LLK-C, LLK, GLQ and NEW-C to a sequence of cell images that records the vasculogenesis process. The sequence has 100 images, and each image has 128×128 pixels. A detailed description of the data can be found in Svoboda et al. [29]. The 1st, 50th and 100th images of the sequence are shown in Figure 5.

In the image denoising literature, to test the noise removal ability of a image denoising method, it is a common practice to add random noise at a certain level to the test images and then apply the image denoising method to the noisy test images (cf., Gijbels et al. [15]). To follow this convention, spatio-temporally correlated noise is first generated using the R-package neuRosim and then added to the sequence of 100 cell images described above. When generating the noise, σ is chosen to be 0.1, 0.2 or 0.3 and ρ is chosen to be 0.1, 0.3 or 0.5, as in the simulation examples presented above. The MSE and EP values of the five image denoising methods based on 10 replicated simulations are presented in Table 3. From the table, it can be seen that NEW still has smaller MSE and EP values in this example, compared to the four competing methods, except in a small number of cases when σ and ρ are relatively small.

**Figure 6:**
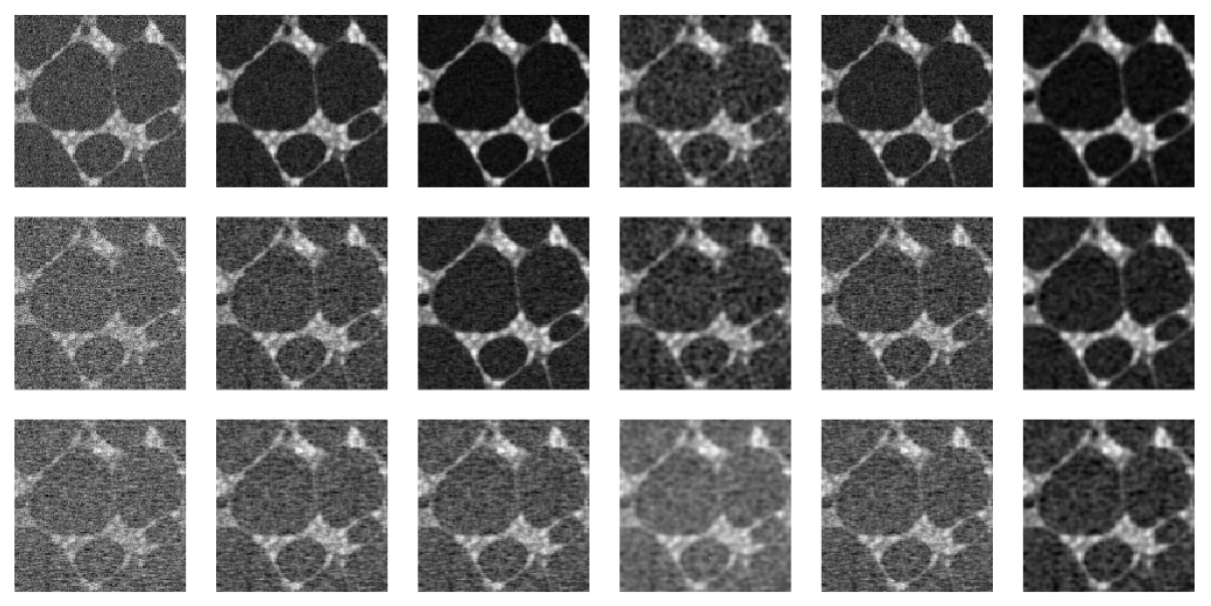
First column shows the 50th observed cell image after the spatio-temporally correlated noise with ρ=0.1 (1st row), 0.3 (2nd row) or 0.5 (3rd row) being added. The second to sixth columns show the denoised images by LLK-C, LLK, GLQ, NEW-C and NEW, respectively.

The 50th observed test image after the spatio-temporally correlated noise with ρ=0.1, 0.3 or 0.5 being added is shown in the first column of Figure 6. The denoised images by the five methods LLK-C, LLK, GLQ, NEW-C and NEW are shown in columns 2–6 of the figure. It can be seen that similar conclusions to those from Figure 4 can be made here, and the denoised images by NEW look reasonably well, as the algorithm work well in removing noise and preserving edges.

**Figure 7:**
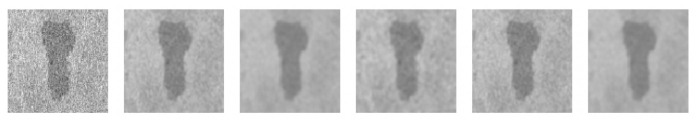
The first image is the observed landsat image of the Salton Sea region taken on 28 April 2001 after the spatio-temporally correlated noise with σ=0.3 and ρ=0.3 being added. Second to sixth images are its denoised versions by LLK-C, LLK, GLQ, NEW-C, and NEW, respectively.

Finally, we apply the five methods considered in the above examples to a sequence of Landsat images of the Salton Sea region. The Salton Sea is the largest inland lake located at the southern border of California, US, and has a great impact on the local ecosystem (Shuford et al. [30]). The Landsat images used here were taken during the time period of 27 May 2000 and 24 December 2001. There are a total of 20 images collected at roughly equally-spaced time points, and each image has 100×100 pixels. In this example, we consider the case when σ=0.3 and ρ=0.3. The MSE values of the five methods LLK-C, LLK, GLQ, NEW-C, and NEW calculated in the same way as before are 9.70, 4.78, 12.03, 9.77, and 4.82, respectively. Their EP values are respectively 85.54%, 20.18%, 109.91%, 86.15%, and 19.14%. So, we can see that NEW method has the best edge-preserving performance among the five methods in this example, and NEW and LLK have the best overall noise removal performance. The 10th noisy observed test image taken on 28 April 2001 and its denoised versions by the five methods are shown in Figure 7. It can be seen from the figure that the denoised images by the methods LLK-C, GLQ, and NEW-C are still quite noisy, and the noise in the images generated by NEW and LLK is mostly removed while the edges are preserved reasonably well.

## 4. Conclusions

In this paper, we have described our proposed edge-preserving image denoising method for handling image sequences. Some major features of the proposed method include (i) helpful information in neighboring images is shared during image denoising, (ii) edge structures in the observed images can be preserved when removing noise, and (iii) possible sptio-temporal data correlation can be accommodated in the related local smoothing procedure. Theoretical arguments given in Section 3.1 and numerical studies presented in Section 3.2 show that the proposed method works well in various cases considered. There are still some issues about the proposed method for future research. For instance, in the proposed local smoothing procedure (2)–(6), each of the bandwidths $\left(h_{x},h_{y},h_{t}\right)$ is chosen by the modified CV procedure (7) and (8) to be the same in the entire design space Ω×[0,1]. Intuitively, relatively small bandwidths are preferred at places where the image intensity surface f(x,y;t) has large curvature and relatively large bandwidths are preferred at places where the curvature of f(x,y;t) is small. Thus, in some applications where the curvature of f(x,y;t) could change quite dramatically in the design space, variable bandwidths might be helpful. Such issues will be studied carefully in our future research.

## Figures and Tables

**Table 1:** In each entry, MSE of $\hat{f}\left(x,y;t\right)$ in (6) is presented in the first line with its standard error (in parenthesis); the corresponding values of $\left(h_{x},h_{t},u\right)$ chosen by the modified CV procedure (7) and (8) is presented in the second line; the optimal MSE is presented in the third line with its standard error (in parenthesis); the optimal values of $\left(h_{xy},h_{t},u\right)$ are presented in the fourth line. MSE in the table has been multiplied by $10^{3}$ and standard error has been multiplied by $10^{5}$.

		$n_{t}=50$		$n_{t}=100$	
σ	ρ	$n_{x}=64$	$n_{x}=128$	$n_{x}=64$	$n_{x}=128$
0.1	0.1	0.65(0.80)	0.30(0.25)	0.48(0.43)	0.26(0.10)
		(0.03, 0.10, 0.05)	(0.03, 0.08, 0.025)	(0.03, 0.10, 0.05)	(0.02, 0.07, 0.05)
		0.32(0.46)	0.20(0.14)	0.37(0.36)	0.19(0.08)
		(0.04, 0.07, 0.025)	(0.03, 0.05, 0.025)	(0.03, 0.08, 0.025)	(0.02, 0.05, 0.025)
	0.3	0.60(0.45)	0.33(0.16)	0.59(0.39)	0.33(0.15)
		(0.04, 0.10, 0.05)	(0.03, 0.07, 0.025)	(0.03, 0.10, 0.05)	(0.02, 0.07, 0.025)
		0.49(0.35)	0.30(0.16)	0.50(0.37)	0.29(0.22)
		(0.04, 0.08, 0.025)	(0.03, 0.06, 0.025)	(0.03, 0.08, 0.025)	(0.03, 0.04, 0.025)
	0.5	1.25(1.24)	0.80(0.22)	0.81(0.55)	0.64(0.21)
		(0.03, 0.10, 0.05)	(0.02, 0.07, 0.025)	(0.03, 0.10, 0.05)	(0.02, 0.04, 0.025)
		0.77(0.65)	0.49(0.24)	0.74(0.46)	0.45(0.25)
		(0.04, 0.09, 0.025)	(0.03, 0.06, 0.025)	(0.03, 0.09, 0.025)	(0.03, 0.04, 0.025)
0.2	0.1	1.14(1.13)	0.68(0.38)	1.02(0.74)	0.56(0.26)
		(0.04, 0.10, 0.025)	(0.03, 0.08, 0.025)	(0.04, 0.10, 0.025)	(0.03, 0.07, 0.025)
		1.11(0.86)	0.66(0.33)	0.93(0.71)	0.54(0.31)
		(0.04, 0.09, 0.025)	(0.03, 0.07, 0.025)	(0.04, 0.08, 0.025)	(0.03, 0.05, 0.025)
	0.3	1.69(0.91)	1.03(0.54)	1.32(1.08)	0.78(0.41)
		(0.04, 0.10, 0.025)	(0.03, 0.08, 0.025)	(0.04, 0.10, 0.025)	(0.03, 0.07, 0.025)
		1.69(1.24)	1.03(0.54)	1.29(1.12)	0.78(0.41)
		(0.04, 0.11, 0.025)	(0.03, 0.08, 0.025)	(0.04, 0.09, 0.025)	(0.03, 0.07, 0.025)
	0.5	3.25(1.74)	2.88(0.78)	1.95(1.85)	2.61(0.58)
		(0.04, 0.07, 0.025)	(0.02, 0.07, 0.025)	(0.04, 0.09, 0.025)	(0.02, 0.04, 0.025)
		2.59(2.23)	1.54(1.32)	1.91(1.78)	1.21(0.43)
		(0.05, 0.10, 0.025)	(0.04, 0.09, 0.025)	(0.04, 0.11, 0.025)	(0.03, 0.08, 0.025)
0.3	0.1	2.32(1.91)	1.26(1.03)	1.59(0.81)	0.92(0.34)
		(0.05, 0.13, 0.025)	(0.04, 0.09, 0.025)	(0.04, 0.11, 0.025)	(0.03, 0.08, 0.025)
		2.28(2.58)	1.26(1.03)	1.59(0.65)	0.92(0.34)
		(0.05, 0.11, 0.025)	(0.04, 0.09, 0.025)	(0.04, 0.10, 0.025)	(0.03, 0.08, 0.025)
	0.3	3.15(2.28)	1.72(1.37)	2.26(1.53)	1.36(0.50)
		(0.05, 0.13, 0.025)	(0.04, 0.09, 0.025)	(0.04, 0.11, 0.025)	(0.03, 0.08, 0.025)
		3.14(2.45)	1.71(1.52)	2.21(1.31)	1.33(0.41)
		(0.05, 0.14, 0.025)	(0.04, 0.10, 0.025)	(0.04, 0.13, 0.025)	(0.04, 0.09, 0.025)
	0.5	6.78(3.46)	6.81(2.00)	4.18(2.72)	6.33(1.43)
		(0.04, 0.09, 0.05)	(0.02, 0.07, 0.05)	(0.04, 0.10, 0.025)	(0.02, 0.04, 0.05)
		4.46(4.94)	2.48(2.38)	3.18(3.42)	1.88(0.56)
		(0.06, 0.16, 0.025)	(0.05, 0.11, 0.025)	(0.05, 0.14, 0.025)	(0.04, 0.10, 0.025)

**Table 2:** In each entry, the first line is the MSE value with its standard error (in parenthesis), and the second line is the EP value. MSE values in the table are in the unit of $10^{3}$ and the standard error values are in the unit of $10^{5}$.

σ	ρ	LLK-C	LLK	GLQ	NEW-C	NEW
0.1	0.1	2.06(0.08)	2.10(0.06)	0.60(0.18)	0.24(0.11)	0.26(0.10)
		73.68%	18.43%	28.24%	12.32%	7.48%
	0.3	3.04(0.14)	2.28(0.09)	0.95(0.18)	2.93(0.40)	0.33(0.15)
		124.48%	34.40%	43.69%	131.28%	10.58%
	0.5	3.89(0.24)	3.23(0.21)	1.42(0.42)	3.77(0.48)	0.64(0.21)
		141.47%	95.86%	57.40%	148.17%	28.86%
0.2	0.1	4.16(0.25)	2.93(0.15)	1.51(0.38)	0.86(0.25)	0.56(0.26)
		142.65%	51.78%	54.40%	39.01%	9.14%
	0.3	9.39(0.52)	3.67(0.25)	2.87(0.51)	9.60(0.78)	0.78(0.41)
		291.31%	82.84%	94.59%	295.72%	15.08%
	0.5	12.80(0.94)	11.21(0.86)	7.75(1.32)	13.12(1.16)	2.61(0.58)
		326.38%	289.71%	203.86%	334.62%	84.24%
0.3	0.1	7.88(0.57)	3.94(0.26)	3.17(0.86)	1.01(0.37)	0.92(0.34)
		235.43%	82.24%	73.18%	23.36%	15.41%
	0.3	19.97(1.15)	5.56(0.50)	12.36(0.63)	19.97(1.16)	1.36(0.50)
		461.12%	133.33%	261.31%	461.13%	25.78%
	0.5	27.64(2.09)	23.75(1.92)	15.75(1.71)	28.04(2.29)	6.33(1.43)
		514.22%	458.82%	292.50%	518.16%	144.58%

**Table 3:** Results for denoising a sequence of 100 cell images. In each entry, the first line is the MSE value and its standard error (in parenthesis), and the second line is the EP value. MSE values in the table are in the unit of $10^{3}$ and the standard errors are in the unit of $10^{5}$.

σ	ρ	LLK-C	LLK	GLQ	NEW-C	NEW
0.1	0.1	1.69(0.11)	0.97(0.08)	1.67(0.12)	1.69(0.12)	1.35(0.12)
		63.30%	5.53%	18.88%	63.31%	18.52%
	0.3	2.36(0.16)	1.43(0.14)	1.94(0.18)	2.36(0.16)	1.51(0.19)
		77.54%	31.64%	25.72%	77.55%	7.28%
	0.5	3.21(0.25)	2.82(0.24)	2.28(0.29)	3.21(0.25)	1.92(0.31)
		88.68%	75.95%	30.68%	88.68%	10.11%
0.2	0.1	3.22(17.00)	1.47(5.54)	3.93(0.29)	3.22(17.00)	1.67(0.25)
		85.64%	13.57%	76.53%	85.64%	16.28%
	0.3	8.71(0.56)	2.34(0.35)	5.00(0.43)	8.71(0.56)	2.17(0.45)
		189.74%	42.07%	91.44%	189.75%	4.88%
	0.5	12.12(0.94)	10.35(0.88)	6.41(0.86)	12.14(0.96)	4.48(0.90)
		213.90%	187.93%	102.68%	214.07%	59.86%
0.3	0.1	3.16(0.50)	2.01(0.28)	5.47(0.53)	3.16(0.50)	1.93(0.40)
		47.15%	22.46%	54.20%	47.15%	10.91%
	0.3	19.30(1.23)	4.29(0.71)	10.11(0.85)	19.30(1.23)	2.82(0.77)
		308.32%	79.75%	161.91%	308.32%	14.37%
	0.5	26.96(2.09)	22.88(1.95)	13.36(1.82)	27.00(2.13)	8.75(1.85)
		345.91%	306.28%	180.35%	346.14%	113.48%

## Data Availability

Publicly available datasets were analyzed in this study. They can be found from the links: https://cbia.fi.muni.cz/datasets/ and https://earthexplorer.usgs.gov.

## Appendix A

### Appendix A.1. Proof of Proposition 1

Define $B_{h}\left(x,y,t\right)=\{\left(x^{{}^{\prime }},y^{{}^{\prime }};t^{{}^{\prime }}\right):\sqrt{\left(\right|x^{{}^{\prime }}-x|/h_{x}{)}^{2}+\left(\right|y^{{}^{\prime }}-y{\left|/h_{y}\right)}^{2}}\leq 1,|t-t^{{}^{\prime }}\left|\leq h_{t},\left(x^{{}^{\prime }},y^{{}^{\prime }};t^{{}^{\prime }}\right)\in \left[0,1\right]\times \left[0,1\right]\times \left[0,1\right]\right\}$, $\Delta_{ijk}=\left[x_{i-1},x_{i}\right]\times \left[y_{j-1},y_{j}\right]\times \left[t_{k-1},t_{k}\right]$, $x_{0}=y_{0}=t_{0}=0$. Then it can be seen that

$$\begin{array}{ccc} & & \left|\frac{1}{NH}\sum_{ijk}K\left(\frac{x_{i}-x}{h_{x}},\frac{y_{i}-y}{h_{y}}\right)K\left(\frac{t_{k}-t}{h_{t}}\right)-1\right|\\ & = & \left|\frac{1}{H}\sum_{ijk}\int \int \int_{\Delta_{ijk}}K\left(\frac{x_{i}-x}{h_{x}},\frac{y_{i}-y}{h_{y}}\right)K\left(\frac{t_{k}-t}{h_{t}}\right)dudvdw-1\right|\\ & = & |\frac{1}{H}\sum_{ijk}\int \int \int_{\Delta_{ijk}}K\left(\frac{x_{i}-x}{h_{x}},\frac{y_{i}-y}{h_{y}}\right)K\left(\frac{t_{k}-t}{h_{t}}\right)dudvdw-\\ & & \frac{1}{H}\int \int \int_{B_{h}\left(x,y,t\right)}K\left(\frac{u-x}{h_{x}},\frac{v-y}{h_{y}}\right)K\left(\frac{w-t}{h_{t}}\right)dudvdw|\\ & = & |\frac{1}{H}\sum_{ijk}\int \int \int_{\Delta_{ijk}}K\left(\frac{x_{i}-x}{h_{x}},\frac{y_{i}-y}{h_{y}}\right)K\left(\frac{t_{k}-t}{h_{t}}\right)dudvdw-\\ & & \frac{1}{H}\sum_{ijk}\int \int \int_{B_{h}\left(x,y,t\right)\cap \Delta_{ijk}}K\left(\frac{u-x}{h_{x}},\frac{v-y}{h_{y}}\right)K\left(\frac{w-t}{h_{t}}\right)dudvdw|\\ & = & |\frac{1}{H}\sum_{ijk}\int \int \int_{B_{h}{\left(x,y,t\right)}^{c}\cap \Delta_{ijk}}K\left(\frac{x_{i}-x}{h_{x}},\frac{y_{i}-y}{h_{y}}\right)K\left(\frac{t_{k}-t}{h_{t}}\right)dudvdw+\\ & & \frac{1}{H}\sum_{ijk}\int \int \int_{B_{h}\left(x,y,t\right)\cap \Delta_{ijk}}K\left(\frac{x_{i}-x}{h_{x}},\frac{y_{i}-y}{h_{y}}\right)K\left(\frac{t_{k}-t}{h_{t}}\right)dudvdw-\\ & & \frac{1}{H}\sum_{ijk}\int \int \int_{B_{h}\left(x,y,t\right)\cap \Delta_{ijk}}K\left(\frac{u-x}{h_{x}},\frac{v-y}{h_{y}}\right)K\left(\frac{w-t}{h_{t}}\right)dudvdw|\\ & \leq & O\left(\frac{1}{n_{min}h_{min}}\right)+\frac{1}{H}\sum_{ijk}\int \int \int_{B_{h}\left(x,y,t\right)\cap \Delta_{ijk}}|K\left(\frac{x_{i}-x}{h_{x}},\frac{y_{j}-y}{h_{y}}\right)K\left(\frac{t_{k}-t}{h_{t}}\right)-\\ & & K\left(\frac{u-x}{h_{x}},\frac{v-y}{h_{y}}\right)K\left(\frac{w-t}{h_{t}}\right)|dudvdw\\ & \leq & O\left(\frac{1}{n_{min}h_{min}}\right)+\frac{1}{H}\sum_{ijk}\int \int \int_{B_{h}\left(x,y,t\right)\cap \Delta_{ijk}}\frac{(1+\sqrt{2})C}{n_{min}h_{min}}dudvdw\\ & = & O\left(\frac{1}{n_{min}h_{min}}\right)+\frac{1}{H}\frac{(1+\sqrt{2})C}{n_{min}h_{min}}\int \int \int_{B_{h}\left(x,y,t\right)}1dudvdw\\ & = & O(\frac{1}{n_{min}h_{min}}), \end{array}$$

where C≥0 is the Lipschitz constant that satisfies the condition $|K\left(u\right)-K\left(u^{{}^{\prime }}\right)|\leq C|u-u^{{}^{\prime }}|$. So, the first result in Proposition 1 is valid.

To prove the second result, it can be checked that

$$\begin{array}{ccc} & & E|\frac{1}{NH}\sum_{ijk}\varepsilon_{ijk}K\left(\frac{x_{i}-x}{h_{x}},\frac{y_{i}-y}{h_{y}}\right)K\left(\frac{t_{i}-x}{h_{t}}\right){|}^{2}\\ & = & Var(\frac{1}{NH}\sum_{ijk}\varepsilon_{ijk}K\left(\frac{x_{i}-x}{h_{x}},\frac{y_{i}-y}{h_{y}}\right)K\left(\frac{t_{k}-x}{h_{t}}\right))\\ & = & \frac{1}{N^{2}H^{2}}\sum_{ijk}\sum_{i^{{}^{\prime }}j^{{}^{\prime }}k^{{}^{\prime }}}K\left(\frac{x_{i}-x}{h_{x}},\frac{y_{i}-y}{h_{y}}\right)K\left(\frac{t_{k}-x}{h_{t}}\right)\\ & & K\left(\frac{x_{i^{{}^{\prime }}}-x}{h_{x}},\frac{y_{i^{{}^{\prime }}}-y}{h_{y}}\right)K\left(\frac{t_{k^{{}^{\prime }}}-x}{h_{t}}\right)Cov\left(\varepsilon_{ijk},\varepsilon_{i^{{}^{\prime }}j^{{}^{\prime }}k^{{}^{\prime }}}\right)\\ & \leq & \frac{1}{N^{2}H^{2}}\sum_{ijk}\sum_{i^{{}^{\prime }}j^{{}^{\prime }}k^{{}^{\prime }}}K\left(\frac{x_{i}-x}{h_{x}},\frac{y_{i}-y}{h_{y}}\right)K\left(\frac{t_{k}-x}{h_{t}}\right)\\ & & K\left(\frac{x_{i^{{}^{\prime }}}-x}{h_{x}},\frac{y_{i^{{}^{\prime }}}-y}{h_{y}}\right)K\left(\frac{t_{k^{{}^{\prime }}}-x}{h_{t}}\right)c_{1}\sigma^{2}\rho^{c_{2}\max\{|i-i^{{}^{\prime }}|,|j-j^{{}^{\prime }}|,|k-k^{{}^{\prime }}\left|\right\}}\\ & \leq & \frac{1}{N^{2}H^{2}}\sum_{ijk}K\left(\frac{x_{i}-x}{h_{x}},\frac{y_{i}-y}{h_{y}}\right)K\left(\frac{t_{k}-x}{h_{t}}\right)c_{1}\sigma^{2}24\int_{0}^{\infty }\tau^{2}\rho^{\tau }d\tau \\ & = & O(\frac{1}{NH}). \end{array}$$

Similarly, it can be checked that

$$\begin{array}{ccc} & & E|\frac{1}{NH}\sum_{ijk}\left(\varepsilon_{ijk}^{2}-\sigma^{2}\right)K\left(\frac{x_{i}-x}{h_{x}},\frac{y_{i}-y}{h_{y}}\right)K\left(\frac{t_{i}-x}{h_{t}}\right){|}^{2}\\ & = & Var(\frac{1}{NH}\sum_{ijk}\varepsilon_{ijk}^{2}K\left(\frac{x_{i}-x}{h_{x}},\frac{y_{i}-y}{h_{y}}\right)K\left(\frac{t_{k}-x}{h_{t}}\right))\\ & = & \frac{1}{N^{2}H^{2}}\sum_{ijk}\sum_{i^{{}^{\prime }}j^{{}^{\prime }}k^{{}^{\prime }}}K\left(\frac{x_{i}-x}{h_{x}},\frac{y_{i}-y}{h_{y}}\right)K\left(\frac{t_{k}-x}{h_{t}}\right)\\ & & K\left(\frac{x_{i^{{}^{\prime }}}-x}{h_{x}},\frac{y_{i^{{}^{\prime }}}-y}{h_{y}}\right)K\left(\frac{t_{k^{{}^{\prime }}}-x}{h_{t}}\right)Cov\left(\varepsilon_{ijk}^{2},\varepsilon_{i^{{}^{\prime }}j^{{}^{\prime }}k^{{}^{\prime }}}^{2}\right)\\ & \leq & \frac{1}{N^{2}H^{2}}\sum_{ijk}\sum_{i^{{}^{\prime }}j^{{}^{\prime }}k^{{}^{\prime }}}K\left(\frac{x_{i}-x}{h_{x}},\frac{y_{i}-y}{h_{y}}\right)K\left(\frac{t_{k}-x}{h_{t}}\right)\\ & & K\left(\frac{x_{i^{{}^{\prime }}}-x}{h_{x}},\frac{y_{i^{{}^{\prime }}}-y}{h_{y}}\right)K\left(\frac{t_{k^{{}^{\prime }}}-x}{h_{t}}\right)12{\left(c_{1}\sigma^{2}\rho^{c_{2}\max\{|i-i^{{}^{\prime }}|,|j-j^{{}^{\prime }}|,|k-k^{{}^{\prime }}\left|\right\}}\right)}^{1/4}E\left(\varepsilon_{111}^{4}\right)\\ & \leq & \frac{1}{N^{2}H^{2}}\sum_{ijk}K\left(\frac{x_{i}-x}{h_{x}},\frac{y_{i}-y}{h_{y}}\right)K\left(\frac{t_{k}-x}{h_{t}}\right)12{\left(c_{1}\sigma^{2}24\int_{0}^{\infty }\tau^{2}\rho^{\tau }d\tau \right)}^{1/3}{\left(E\left(\varepsilon_{111}^{6}\right)\right)}^{2/3}\\ & = & O(\frac{1}{NH}). \end{array}$$

The first inequality in the above expression is based on the result in Davydov [31]. So, the third result is valid.

### Appendix A.2. Proof of Theorem 1

We first consider the case when $\left(x,y;t\right)\in \Omega_{h}\setminus J_{h}$. By Taylor expansion, we have

$$\begin{array}{cc} Z_{ijk}= & f\left(x_{i},y_{j};t_{k}\right)+\epsilon_{ijk}\\ = & f\left(x,y;t\right)+\left(x_{i}-x\right)f_{x}^{{}^{\prime }}\left(x,y;t\right)+\left(y_{j}-y\right)f_{y}^{{}^{\prime }}\left(x,y;t\right)+\left(t_{k}-t\right)f_{t}^{{}^{\prime }}\left(x,y;t\right)+\\ \; & O\left(h_{x}^{2}+h_{y}^{2}+h_{t}^{2}\right)+\epsilon_{ijk}. \end{array}$$

So, it can be checked that

$$\begin{array}{cc} \left[\begin{array}{c} \sum_{ijk}Z_{ijk}K_{ijk}\\ \sum_{ijk}\left(x_{i}-x\right)Z_{ijk}K_{ijk}\\ \sum_{ijk}\left(y_{j}-y\right)Z_{ijk}K_{ijk}\\ \sum_{ijk}\left(t_{k}-t\right)Z_{ijk}K_{ijk} \end{array}\right]= & M\left[\begin{array}{c} f(x,y;t)\\ f_{x}^{{}^{\prime }}\left(x,y;t\right)\\ f_{y}^{{}^{\prime }}\left(x,y;t\right)\\ f_{t}^{{}^{\prime }}\left(x,y;t\right) \end{array}\right]+\left[\begin{array}{c} \sum_{ijk}O\left(h_{x}^{2}+h_{y}^{2}+h_{t}^{2}\right)K_{ijk}\\ \sum_{ijk}\left(x_{i}-x\right)O\left(h_{x}^{2}+h_{y}^{2}+h_{t}^{2}\right)K_{ijk}\\ \sum_{ijk}\left(y_{j}-y\right)O\left(h_{x}^{2}+h_{y}^{2}+h_{t}^{2}\right)K_{ijk}\\ \sum_{ijk}\left(t_{k}-t\right)O\left(h_{x}^{2}+h_{y}^{2}+h_{t}^{2}\right)K_{ijk} \end{array}\right]+\\ \; & \left[\begin{array}{c} \sum_{ijk}\epsilon_{ijk}K_{ijk}\\ \sum_{ijk}\left(x_{i}-x\right)\epsilon_{ijk}K_{ijk}\\ \sum_{ijk}\left(y_{j}-y\right)\epsilon_{ijk}K_{ijk}\\ \sum_{ijk}\left(t_{k}-t\right)\epsilon_{ijk}K_{ijk} \end{array}\right], \end{array}$$

where

$$\begin{array}{c} M=\left[\begin{array}{cccc} m_{000} & m_{100} & m_{010} & m_{001}\\ m_{100} & m_{200} & m_{110} & m_{101}\\ m_{010} & m_{110} & m_{020} & m_{011}\\ m_{001} & m_{101} & m_{011} & m_{002} \end{array}\right]. \end{array}$$

From Expression (3), we have

$$\begin{array}{cc} \left[\begin{array}{c} \hat{a}\left(x,y;t\right)\\ \hat{b}\left(x,y;t\right)\\ \hat{c}\left(x,y;t\right)\\ \hat{d}\left(x,y;t\right) \end{array}\right]= & \left[\begin{array}{c} f(x,y;t)\\ f_{x}^{{}^{\prime }}\left(x,y;t\right)\\ f_{y}^{{}^{\prime }}\left(x,y;t\right)\\ f_{t}^{{}^{\prime }}\left(x,y;t\right) \end{array}\right]+M^{-1}\left[\begin{array}{c} \sum_{ijk}O\left(h_{x}^{2}+h_{y}^{2}+h_{t}^{2}\right)K_{ijk}\\ \sum_{ijk}\left(x_{i}-x\right)O\left(h_{x}^{2}+h_{y}^{2}+h_{t}^{2}\right)K_{ijk}\\ \sum_{ijk}\left(y_{j}-y\right)O\left(h_{x}^{2}+h_{y}^{2}+h_{t}^{2}\right)K_{ijk}\\ \sum_{ijk}\left(t_{k}-t\right)O\left(h_{x}^{2}+h_{y}^{2}+h_{t}^{2}\right)K_{ijk} \end{array}\right]+\\ \; & M^{-1}\left[\begin{array}{c} \sum_{ijk}\epsilon_{ijk}K_{ijk}\\ \sum_{ijk}\left(x_{i}-x\right)\epsilon_{ijk}K_{ijk}\\ \sum_{ijk}\left(y_{j}-y\right)\epsilon_{ijk}K_{ijk}\\ \sum_{ijk}\left(t_{k}-t\right)\epsilon_{ijk}K_{ijk} \end{array}\right]. \end{array}$$

By some simple algebraic manipulations, we have

$$\begin{array}{cc} M^{-1}= & \left[\begin{array}{cccc} O(\frac{1}{NH}) & O(\frac{1}{NH\cdot h_{x}}) & O(\frac{1}{NH\cdot h_{y}}) & O(\frac{1}{NH\cdot h_{t}})\\ O(\frac{1}{NH\cdot h_{x}}) & O(\frac{1}{NH\cdot h_{x}^{2}}) & O(\frac{1}{NH\cdot h_{x}h_{y}}) & O(\frac{1}{NH\cdot h_{x}h_{t}})\\ O(\frac{1}{NH\cdot h_{y}}) & O(\frac{1}{NH\cdot h_{x}h_{y}}) & O(\frac{1}{NH\cdot h_{y}^{2}}) & O(\frac{1}{NH\cdot h_{y}h_{t}})\\ O(\frac{1}{NH\cdot h_{t}}) & O(\frac{1}{NH\cdot h_{x}h_{t}}) & O(\frac{1}{NH\cdot h_{y}h_{t}}) & O(\frac{1}{NH\cdot h_{t}^{2}}) \end{array}\right]. \end{array}$$

Then,

$$\begin{array}{cc} \left[\begin{array}{c} \hat{a}\left(x,y;t\right)\\ \hat{b}\left(x,y;t\right)\\ \hat{c}\left(x,y;t\right)\\ \hat{d}\left(x,y;t\right) \end{array}\right]= & \left[\begin{array}{c} f(x,y;t)\\ f_{x}^{{}^{\prime }}\left(x,y;t\right)\\ f_{y}^{{}^{\prime }}\left(x,y;t\right)\\ f_{t}^{{}^{\prime }}\left(x,y;t\right) \end{array}\right]+\left[\begin{array}{c} O(h_{x}^{2}+h_{y}^{2}+h_{t}^{2})\\ O(\frac{h_{x}^{2}+h_{y}^{2}+h_{t}^{2}}{h_{x}})\\ O(\frac{h_{x}^{2}+h_{y}^{2}+h_{t}^{2}}{h_{y}})\\ O(\frac{h_{x}^{2}+h_{y}^{2}+h_{t}^{2}}{h_{t}}) \end{array}\right]+\left[\begin{array}{c} O_{p}\left(\frac{1}{\sqrt{NH}}\right)\\ O_{p}\left(\frac{1}{h_{x}\sqrt{NH}}\right)\\ O_{p}\left(\frac{1}{h_{y}\sqrt{NH}}\right)\\ O_{p}\left(\frac{1}{h_{t}\sqrt{NH}}\right) \end{array}\right]. \end{array}$$

Now, we consider the case when $\left(x,y;t\right)\in J_{h}\setminus S_{h}$. If $\left(x_{i},y_{j};t_{k}\right)\in I_{1}$, then we have

$$\begin{array}{ccc} Z_{ijk} & = & f\left(x_{i},y_{j};t_{k}\right)+\varepsilon_{ijk}\\ & = & f_{-}\left(x_{\tau },y_{\tau };t_{\tau }\right)+O\left(\sqrt{h_{x}^{2}+h_{y}^{2}+h_{t}^{2}}\right)+\varepsilon_{ijk}, \end{array}$$

and if $\left(x_{i},y_{j};t_{k}\right)\in I_{2}$, we have

$$\begin{array}{ccc} Z_{ijk} & = & f\left(x_{i},y_{j};t_{k}\right)+\varepsilon_{ijk}\\ & = & f_{-}\left(x_{\tau },y_{\tau };t_{\tau }\right)+d_{\tau }+O\left(\sqrt{h_{x}^{2}+h_{y}^{2}+h_{t}^{2}}\right)+\varepsilon_{ijk}. \end{array}$$

By some similar arguments to those in the case considered above, we have

$$\begin{array}{ccc} \left[\begin{array}{c} \hat{a}\left(x,y;t\right)\\ \hat{b}\left(x,y;t\right)\\ \hat{c}\left(x,y;t\right)\\ \hat{d}\left(x,y;t\right) \end{array}\right] & = & \left[\begin{array}{c} f_{-}\left(x_{\tau },y_{\tau };t_{\tau }\right)+d_{\tau }\frac{\sum_{\left(x_{i},y_{j};t_{k}\right)\in I_{2}}K_{ijk}}{\sum_{ijk}K_{ijk}}\\ \frac{d_{\tau }}{h_{x}}\frac{\sum_{\left(x_{i},y_{j};t_{k}\right)\in I_{2}}\left[\left(x_{i}-x\right)/h_{x}\right]K_{ijk}}{\sum_{ijk}{\left[\left(x_{i}-x\right)/h_{x}\right]}^{2}K_{ijk}}\\ \frac{d_{\tau }}{h_{y}}\frac{\sum_{\left(x_{i},y_{j};t_{k}\right)\in I_{2}}\left[\left(y_{j}-y\right)/h_{y}\right]K_{ijk}}{\sum_{ijk}{\left[\left(y_{j}-y\right)/h_{y}\right]}^{2}K_{ijk}}\\ \frac{d_{\tau }}{h_{t}}\frac{\sum_{\left(x_{i},y_{j};t_{k}\right)\in I_{2}}\left[\left(t_{k}-t\right)/h_{t}\right]K_{ijk}}{\sum_{ijk}{\left[\left(t_{k}-t\right)/h_{t}\right]}^{2}K_{ijk}} \end{array}\right]+\\ & & \left[\begin{array}{c} O(\sqrt{h_{x}^{2}+h_{y}^{2}+h_{t}^{2}})\\ O(\frac{\sqrt{h_{x}^{2}+h_{y}^{2}+h_{t}^{2}}}{h_{x}})\\ O(\frac{\sqrt{h_{x}^{2}+h_{y}^{2}+h_{t}^{2}}}{h_{y}})\\ O(\frac{\sqrt{h_{x}^{2}+h_{y}^{2}+h_{t}^{2}}}{h_{t}}) \end{array}\right]+\left[\begin{array}{c} O_{p}\left(\frac{1}{\sqrt{NH}}\right)\\ O_{p}\left(\frac{1}{h_{x}\sqrt{NH}}\right)\\ O_{p}\left(\frac{1}{h_{y}\sqrt{NH}}\right)\\ O_{p}\left(\frac{1}{h_{t}\sqrt{NH}}\right) \end{array}\right]\\ & = & \left[\begin{array}{c} f_{-}\left(x_{\tau },y_{\tau };t_{\tau }\right)+d_{\tau }\xi_{000}^{\left(2\right)}\\ \frac{d_{\tau }}{\xi_{200}h_{x}}\xi_{100}^{\left(2\right)}\\ \frac{d_{\tau }}{\xi_{020}h_{y}}\xi_{010}^{\left(2\right)}\\ \frac{d_{\tau }}{\xi_{002}h_{t}}\xi_{001}^{\left(2\right)} \end{array}\right]+\left[\begin{array}{c} O(\sqrt{h_{x}^{2}+h_{y}^{2}+h_{t}^{2}})\\ O(\frac{\sqrt{h_{x}^{2}+h_{y}^{2}+h_{t}^{2}}}{h_{x}})\\ O(\frac{\sqrt{h_{x}^{2}+h_{y}^{2}+h_{t}^{2}}}{h_{y}})\\ O(\frac{\sqrt{h_{x}^{2}+h_{y}^{2}+h_{t}^{2}}}{h_{t}}) \end{array}\right]+\left[\begin{array}{c} O_{p}\left(\frac{1}{\sqrt{NH}}\right)\\ O_{p}\left(\frac{1}{h_{x}\sqrt{NH}}\right)\\ O_{p}\left(\frac{1}{h_{y}\sqrt{NH}}\right)\\ O_{p}\left(\frac{1}{h_{t}\sqrt{NH}}\right) \end{array}\right] \end{array}$$

### Appendix A.3. Proof of Theorem 2

We prove the second equations in (10) and (11) here. The first equations can be proved similarly. For simplicity, we write ${\hat{a}}^{\left(l\right)}\left(x,y;t\right)$, ${\hat{b}}^{\left(l\right)}\left(x,y;t\right)$, ${\hat{c}}^{\left(l\right)}\left(x,y;t\right)$, ${\hat{d}}^{\left(l\right)}\left(x,y;t\right)$, $O^{\left(l\right)}\left(x,y;t\right)$ and ${\tilde{O}}^{\left(l\right)}\left(x,y;t\right)$ as ${\hat{a}}^{\left(l\right)}$, ${\hat{b}}^{\left(l\right)}$, ${\hat{c}}^{\left(l\right)}$, ${\hat{d}}^{\left(l\right)}$, $O^{\left(l\right)}$ and ${\tilde{O}}^{\left(l\right)}$, respectively from now on. First, by Proposition 1, it is easy to show that

(A1) $$\frac{\sum_{ijk}\varepsilon_{ijk}K\left(\frac{x_{i}-x}{h_{x}},\frac{y_{i}-y}{h_{y}}\right)K\left(\frac{t_{i}-x}{h_{t}}\right)}{\sum_{ijk}K\left(\frac{x_{i}-x}{h_{x}},\frac{y_{i}-y}{h_{y}}\right)K\left(\frac{t_{i}-x}{h_{t}}\right)}=O_{p}\left(\frac{1}{\sqrt{NH}}\right),$$

(A2) $$\frac{\sum_{ijk}\left(\varepsilon_{ijk}^{2}-\sigma^{2}\right)K\left(\frac{x_{i}-x}{h_{x}},\frac{y_{i}-y}{h_{y}}\right)K\left(\frac{t_{i}-x}{h_{t}}\right)}{\sum_{ijk}K\left(\frac{x_{i}-x}{h_{x}},\frac{y_{i}-y}{h_{y}}\right)K\left(\frac{t_{i}-x}{h_{t}}\right)}=o_{p}\left(1\right).$$

Let us first consider the case when $\left(x,y;t\right)\in \Omega_{h}\setminus J_{h}$. In such a case, it can be checked that

$$\begin{array}{ccc} e^{\left(l\right)}\left(x,y;t\right) & = & \{\sum_{\left(x_{i},y_{j};t_{k}\right)\in O^{\left(l\right)}}[\varepsilon_{ijk}+f\left(x_{i},y_{j};t_{k}\right)-{\hat{a}}^{\left(l\right)}-{\hat{b}}^{\left(l\right)}\left(x_{i}-x\right)-\\ & & {\hat{c}}^{\left(l\right)}\left(y_{j}-y\right)-{\hat{d}}^{\left(l\right)}\left(t_{k}-t\right){]}^{2}K_{ijk}\}/\sum_{\left(x_{i},y_{j};t_{k}\right)\in O^{\left(l\right)}}K_{ijk}\\ & = & \left\{\sum_{\left(x_{i},y_{j};t_{k}\right)\in O^{\left(l\right)}}\varepsilon_{ijk}^{2}K_{ijk}\right\}/\sum_{\left(x_{i},y_{j};t_{k}\right)\in O^{\left(l\right)}}K_{ijk}+\\ & & \{2\sum_{\left(x_{i},y_{j};t_{k}\right)\in O^{\left(l\right)}}\varepsilon_{ijk}[f\left(x_{i},y_{j};t_{k}\right)-{\hat{a}}^{\left(l\right)}-{\hat{b}}^{\left(l\right)}\left(x_{i}-x\right)-\\ & & {\hat{c}}^{\left(l\right)}\left(y_{j}-y\right)-{\hat{d}}^{\left(l\right)}\left(t_{k}-t\right)]K_{ijk}\}/\sum_{\left(x_{i},y_{j};t_{k}\right)\in O^{\left(l\right)}}K_{ijk}+\\ & & \{\sum_{\left(x_{i},y_{j};t_{k}\right)\in O^{\left(l\right)}}[f\left(x_{i},y_{j};t_{k}\right)-{\hat{a}}^{\left(l\right)}-{\hat{b}}^{\left(l\right)}\left(x_{i}-x\right)-\\ & & {\hat{c}}^{\left(l\right)}\left(y_{j}-y\right)-{\hat{d}}^{\left(l\right)}\left(t_{k}-t\right){]}^{2}K_{ijk}\}/\sum_{\left(x_{i},y_{j};t_{k}\right)\in O^{\left(l\right)}}K_{ijk}\\ & =: & A_{1}^{\left(l\right)}\left(x,y;t\right)+A_{2}^{\left(l\right)}\left(x,y;t\right)+A_{3}^{\left(l\right)}\left(x,y;t\right). \end{array}$$

Similar to (A2), we have

(A3) $$A_{1}^{\left(l\right)}\left(x,y;t\right)=\sigma^{2}+o_{p}\left(1\right).$$

Taylor expansion of $f(x_{i},y_{j};t_{k})$ at point (x,y;t), results in Theorem 1, and by similar arguments for (A1), we have

(A4) $$\begin{array}{ccc} A_{2}^{\left(l\right)}\left(x,y;t\right) & \leq & 2|f\left(x,y;t\right)-{\hat{a}}^{\left(l\right)}\left|\right|\frac{\sum_{\left(x_{i},y_{j};t_{k}\right)\in O^{\left(l\right)}}\varepsilon_{ijk}K_{ijk}}{\sum_{\left(x_{i},y_{j};t_{k}\right)\in O^{\left(l\right)}}K_{ijk}}|+\\ & & 2h_{x}\left|f_{x}^{{}^{\prime }}\left(x,y;t\right)-{\hat{b}}^{\left(l\right)}\right||\frac{\sum_{\left(x_{i},y_{j};t_{k}\right)\in O^{\left(l\right)}}\varepsilon_{ijk}\frac{x_{i}-x}{h_{x}}K_{ijk}}{\sum_{\left(x_{i},y_{j};t_{k}\right)\in O^{\left(l\right)}}K_{ijk}}|+\\ & & 2h_{y}\left|f_{y}^{{}^{\prime }}\left(x,y;t\right)-{\hat{c}}^{\left(l\right)}\right||\frac{\sum_{\left(x_{i},y_{j};t_{k}\right)\in O^{\left(l\right)}\left(x,y;t\right)}\varepsilon_{ijk}\frac{y_{j}-y}{h_{y}}K_{ijk}}{\sum_{\left(x_{i},y_{j};t_{k}\right)\in O^{\left(l\right)}}K_{ijk}}|+\\ & & 2h_{t}\left|f_{t}^{{}^{\prime }}\left(x,y;t\right)-{\hat{d}}^{\left(l\right)}\right||\frac{\sum_{\left(x_{i},y_{j};t_{k}\right)\in O^{\left(l\right)}}\varepsilon_{ijk}\frac{t_{k}-t}{h_{t}}K_{ijk}}{\sum_{\left(x_{i},y_{j};t_{k}\right)\in O^{\left(l\right)}}K_{ijk}}|\\ & = & o_{p}\left(1\right). \end{array}$$

Similarly, we have

(A5) $$\begin{array}{c} A_{3}^{\left(l\right)}\left(x,y;t\right)=o_{p}\left(1\right). \end{array}$$

By combining (A3)–(A5), we have

$$e^{\left(l\right)}\left(x,y;t\right)=\sigma^{2}+o_{p}\left(1\right).$$

Now, let us consider the case when $\left(x,y;t\right)\in J_{h}\setminus S_{h}$. Similar to the above case, let us write

$$e^{\left(l\right)}\left(x,y;t\right)=A_{1}^{\left(l\right)}\left(x,y;t\right)+A_{2}^{\left(l\right)}\left(x,y;t\right)+A_{3}^{\left(l\right)}\left(x,y;t\right).$$

Here, we still have

(A6) $$A_{1}^{\left(l\right)}\left(x,y;t\right)=\sigma^{2}+o_{p}\left(1\right).$$

For $A_{2}^{\left(l\right)}\left(x,y;t\right)$, we have

$$\begin{array}{ccc} A_{2}^{\left(l\right)}\left(x,y;t\right) & = & \{2\sum_{\left(x_{i},y_{j};t_{k}\right)\in I^{1}\cap O^{\left(l\right)}}\varepsilon_{ijk}[f\left(x_{i},y_{j};t_{k}\right)-{\hat{a}}^{\left(l\right)}-{\hat{b}}^{\left(l\right)}\left(x_{i}-x\right)-\\ & & {\hat{c}}^{\left(l\right)}\left(y_{j}-y\right)-{\hat{d}}^{\left(l\right)}\left(t_{k}-t\right)]K_{ijk}\}/\sum_{\left(x_{i},y_{j};t_{k}\right)\in O^{\left(l\right)}}K_{ijk}+\\ & & \{2\sum_{\left(x_{i},y_{j};t_{k}\right)\in I^{2}\cap O^{\left(l\right)}}\varepsilon_{ijk}[f\left(x_{i},y_{j};t_{k}\right)-{\hat{a}}^{\left(l\right)}-{\hat{b}}^{\left(l\right)}\left(x_{i}-x\right)-\\ & & {\hat{c}}^{\left(l\right)}\left(y_{j}-y\right)-{\hat{d}}^{\left(l\right)}\left(t_{k}-t\right)]K_{ijk}\}/\sum_{\left(x_{i},y_{j};t_{k}\right)\in O^{\left(l\right)}}K_{ijk}\\ & =: & A_{21}^{\left(l\right)}\left(x,y;t\right)+A_{22}^{\left(l\right)}\left(x,y;t\right). \end{array}$$

By the results in Theorem 1, we have

$$\begin{array}{ccc} A_{21}^{\left(l\right)}\left(x,y;t\right) & = & \frac{2\sum_{\left(x_{i},y_{j};t_{k}\right)\in I^{1}\cap O^{\left(l\right)}}\varepsilon_{ijk}\left[f\left(x_{i},y_{j};t_{k}\right)-f_{-}\left(x_{\tau },y_{\tau };t_{\tau }\right)\right]K_{ijk}}{\sum_{\left(x_{i},y_{j};t_{k}\right)\in O^{\left(l\right)}}K_{ijk}}-\\ & & \frac{\left(D_{1}+o_{p}\left(1\right)\right)\sum_{\left(x_{i},y_{j};t_{k}\right)\in I^{1}\cap O^{\left(l\right)}}\varepsilon_{ijk}K_{ijk}}{\sum_{\left(x_{i},y_{j};t_{k}\right)\in O^{\left(l\right)}}K_{ijk}}-\\ & & \frac{\left(D_{2}+o_{p}\left(1\right)\right)\sum_{\left(x_{i},y_{j};t_{k}\right)\in I^{1}\cap O^{\left(l\right)}}\varepsilon_{ijk}\frac{x_{i}-x}{h_{x}}K_{ijk}}{\sum_{\left(x_{i},y_{j};t_{k}\right)\in O^{\left(l\right)}}K_{ijk}}-\\ & & \frac{\left(D_{3}+o_{p}\left(1\right)\right)\sum_{\left(x_{i},y_{j};t_{k}\right)\in I^{1}\cap O^{\left(l\right)}}\varepsilon_{ijk}\frac{y_{j}-y}{h_{y}}K_{ijk}}{\sum_{\left(x_{i},y_{j};t_{k}\right)\in O^{\left(l\right)}}K_{ijk}}-\\ & & \frac{\left(D_{4}+o_{p}\left(1\right)\right)\sum_{\left(x_{i},y_{j};t_{k}\right)\in I^{1}\cap O^{\left(l\right)}}\varepsilon_{ijk}\frac{t_{k}-t}{h_{t}}K_{ijk}}{\sum_{\left(x_{i},y_{j};t_{k}\right)\in O^{\left(l\right)}}K_{ijk}}, \end{array}$$

where $D_{1}$, $D_{2}$, $D_{3}$ and $D_{4}$ are constants. By similar arguments for (A1), we can conclude that

$$A_{21}^{\left(l\right)}=o_{p}\left(1\right).$$

Similarly, we have

$$A_{22}^{\left(l\right)}=o_{p}\left(1\right).$$

So,

(A7) $$A_{2}^{\left(l\right)}=o_{p}\left(1\right).$$

By similar arguments to those about Proposition 1, we have

$$|\frac{1}{NH}\sum_{\left(x_{i},y_{j};t_{k}\right)\in O^{\left(l\right)}}K_{ijk}-\frac{1}{2}|=o\left(1\right).$$

For a function ϕ(x,y;t) satisfying the condition that $\sup_{x^{2}+y^{2}+t^{2}\leq 1}\left|\phi \left(x,y;t\right)\right|\leq b_{\phi }<\infty$, we can have

$$\begin{array}{ccc} & & |\frac{1}{NH}\sum_{\left(x_{i},y_{j};t_{k}\right)\in I^{1}⋂O^{\left(l\right)}}\phi \left(\frac{x_{i}-x}{h_{x}},\frac{y_{j}-y}{h_{y}};\frac{t_{k}-t}{h_{t}}\right)K_{ijk}-\\ & & \frac{1}{NH}\sum_{\left(x_{i},y_{j};t_{k}\right)\in I^{1}⋂{\tilde{O}}^{\left(l\right)}}\phi \left(\frac{x_{i}-x}{h_{x}},\frac{y_{j}-y}{h_{y}};\frac{t_{k}-t}{h_{t}}\right)K_{ijk}|\\ & \leq & b_{\phi }\left||K|\right|\frac{1}{NH}\sum_{\left(x_{i},y_{j};t_{k}\right)\in O^{\left(l\right)}\Delta {\tilde{O}}^{\left(l\right)}}1\\ & = & o(1), \end{array}$$

where $O^{\left(l\right)}\Delta {\tilde{O}}^{\left(l\right)}=\left(O^{\left(l\right)}⋃{\tilde{O}}^{\left(l\right)}\right)\setminus \left(O^{\left(l\right)}⋂{\tilde{O}}^{\left(l\right)}\right)$. The last equation above is a direct conclusion of (9). By the above results, we have

(A8) $$\begin{array}{ccc} A_{3}^{\left(l\right)}\left(x,y;t\right) & = & \frac{2}{NH}\sum_{\left(x_{i},y_{j};t_{k}\right)\in O^{\left(l\right)}}[f\left(x_{i},y_{j};t_{k}\right)-{\hat{a}}^{\left(l\right)}-{\hat{b}}^{\left(l\right)}\left(x_{i}-x\right)-\\ & & {\hat{c}}^{\left(l\right)}\left(y_{j}-y\right)-{\hat{d}}^{\left(l\right)}\left(t_{k}-t\right){]}^{2}K_{ijk}\\ & = & \frac{2}{NH}\sum_{\left(x_{i},y_{j};t_{k}\right)\in O^{\left(l\right)}}[f\left(x_{i},y_{j};t_{k}\right)-f_{-}\left(x_{\tau },y_{\tau };t_{\tau }\right)-d_{\tau }B_{0l}-\frac{d_{\tau }B_{1l}}{\xi_{200}}\frac{x_{i}-x}{h_{x}}-\\ & & \frac{d_{\tau }B_{2l}}{\xi_{020}}\frac{y_{j}-y}{h_{y}}-\frac{d_{\tau }B_{3l}}{\xi_{002}}\frac{t_{k}-t}{h_{t}}{]}^{2}K_{ijk}+o_{p}\left(1\right)\\ & = & \frac{2}{NH}\left(\sum_{\left(x_{i},y_{j};t_{k}\right)\in I^{1}\cap O^{\left(l\right)}}+\sum_{\left(x_{i},y_{j};t_{k}\right)\in I^{2}\cap O^{\left(l\right)}}\right)\\ & & [f\left(x_{i},y_{j};t_{k}\right)-f_{-}\left(x_{\tau },y_{\tau };t_{\tau }\right)-d_{\tau }B_{0l}-\frac{d_{\tau }B_{1l}}{\xi_{200}}\frac{x_{i}-x}{h_{x}}-\\ & & \frac{d_{\tau }B_{2l}}{\xi_{020}}\frac{y_{j}-y}{h_{y}}-\frac{d_{\tau }B_{3l}}{\xi_{002}}\frac{t_{k}-t}{h_{t}}{]}^{2}K_{ijk}+o_{p}\left(1\right)\\ & = & \frac{2}{NH}\left(\sum_{\left(x_{i},y_{j};t_{k}\right)\in I^{1}\cap {\tilde{O}}^{\left(l\right)}}+\sum_{\left(x_{i},y_{j};t_{k}\right)\in I^{2}\cap {\tilde{O}}^{\left(l\right)}}\right)\\ & & [f\left(x_{i},y_{j};t_{k}\right)-f_{-}\left(x_{\tau },y_{\tau };t_{\tau }\right)-d_{\tau }B_{0l}-\frac{d_{\tau }B_{1l}}{\xi_{200}}\frac{x_{i}-x}{h_{x}}-\\ & & \frac{d_{\tau }B_{2l}}{\xi_{020}}\frac{y_{j}-y}{h_{y}}-\frac{d_{\tau }B_{3l}}{\xi_{002}}\frac{t_{k}-t}{h_{t}}{]}^{2}K_{ijk}+o_{p}\left(1\right)\\ & = & \frac{2}{NH}\sum_{\left(x_{i},y_{j};t_{k}\right)\in I^{1}\cap {\tilde{O}}^{\left(l\right)}}[-d_{\tau }B_{0l}-\frac{d_{\tau }B_{1l}}{\xi_{200}}\frac{x_{i}-x}{h_{x}}-\\ & & \frac{d_{\tau }B_{2l}}{\xi_{020}}\frac{y_{j}-y}{h_{y}}-\frac{d_{\tau }B_{3l}}{\xi_{002}}\frac{t_{k}-t}{h_{t}}{]}^{2}K_{ijk}+\\ & & \frac{2}{NH}\sum_{\left(x_{i},y_{j};t_{k}\right)\in I^{2}\cap {\tilde{O}}^{\left(l\right)}}[d_{\tau }-d_{\tau }B_{0l}-\frac{d_{\tau }B_{1l}}{\xi_{200}}\frac{x_{i}-x}{h_{x}}-\\ & & \frac{d_{\tau }B_{2l}}{\xi_{020}}\frac{y_{j}-y}{h_{y}}-\frac{d_{\tau }B_{3l}}{\xi_{002}}\frac{t_{k}-t}{h_{t}}{]}^{2}K_{ijk}+o_{p}\left(1\right) \end{array}$$

$$\begin{array}{ccc} & = & 2d_{\tau }^{2}\int \int \int_{Q^{\left(1l\right)}}{\left[B_{0l}+\frac{B_{1l}}{\xi_{200}}u+\frac{B_{2l}}{\xi_{020}}v+\frac{B_{3l}}{\xi_{002}}w\right]}^{2}K\left(u,v\right)K\left(w\right)dudvdw+\\ & & 2d_{\tau }^{2}\int \int \int_{Q^{\left(2l\right)}}{\left[1-B_{0l}-\frac{B_{1l}}{\xi_{200}}u-\frac{B_{2l}}{\xi_{020}}v-\frac{B_{3l}}{\xi_{002}}w\right]}^{2}K\left(u,v\right)K\left(w\right)dudvdw\\ & & +o_{p}\left(1\right)\\ & = & d_{\tau }^{2}{\left(C_{\tau }^{\left(l\right)}\right)}^{2}+o_{p}\left(1\right), \end{array}$$

where

$$\begin{array}{ccc} C_{\tau }^{\left(l\right)} & = & (2\int \int \int_{Q^{\left(1l\right)}}{\left[B_{0l}+\frac{B_{1l}}{\xi_{200}}u+\frac{B_{2l}}{\xi_{020}}v+\frac{B_{3l}}{\xi_{002}}w\right]}^{2}K\left(u,v\right)K\left(w\right)dudvdw+\\ & & 2\int \int \int_{Q^{\left(2l\right)}}{\left[1-B_{0l}-\frac{B_{1l}}{\xi_{200}}u-\frac{B_{2l}}{\xi_{020}}v-\frac{B_{3l}}{\xi_{002}}w\right]}^{2}K\left(u,v\right)K\left(w\right)dudvdw{)}^{1/2}. \end{array}$$

Then by equation (A6)–(A8), we have

$$e^{\left(l\right)}\left(x,y;t\right)=\sigma^{2}+d_{\tau }^{2}{\left(C_{\tau }^{\left(l\right)}\right)}^{2}+o_{p}\left(1\right).$$

Similarly, we can prove that

$$e\left(x,y;t\right)=\sigma^{2}+d_{\tau }^{2}{\left(C_{\tau }\right)}^{2}+o_{p}\left(1\right),$$

where

$$\begin{array}{ccc} C_{\tau } & = & (\int \int \int_{Q^{\left(1\right)}}{\left[\xi_{000}^{\left(2\right)}+\frac{\xi_{100}^{\left(2\right)}}{\xi_{200}}u+\frac{\xi_{010}^{\left(2\right)}}{\xi_{020}}v+\frac{\xi_{001}^{\left(2\right)}}{\xi_{002}}w\right]}^{2}K\left(u,v\right)K\left(w\right)dudvdw+\\ & & \int \int \int_{Q^{\left(2\right)}}{\left[1-\xi_{000}^{\left(2\right)}-\frac{\xi_{100}^{\left(2\right)}}{\xi_{200}}u-\frac{\xi_{010}^{\left(2\right)}}{\xi_{020}}v-\frac{\xi_{001}^{\left(2\right)}}{\xi_{002}}w\right]}^{2}K\left(u,v\right)K\left(w\right)dudvdw{)}^{1/2}. \end{array}$$

The main difference between this case and the previous case in the proof is in the derivation of the result of (A8). For e(x,y;t), the corresponding result is

$$\begin{array}{ccc} A_{3}\left(x,y;t\right) & = & \frac{1}{NH}\sum_{\left(x_{i},y_{j};t_{k}\right)}[f\left(x_{i},y_{j};t_{k}\right)-\hat{a}\left(x,y;t\right)-\hat{b}\left(x,y;t\right)\left(x_{i}-x\right)-\\ & & \hat{c}\left(x,y;t\right)\left(y_{j}-y\right)-\hat{d}\left(x,y;t\right)\left(t_{k}-t\right){]}^{2}K_{ijk}\\ & = & \frac{1}{NH}\sum_{\left(x_{i},y_{j};t_{k}\right)}[f\left(x_{i},y_{j};t_{k}\right)-f_{-}\left(x_{\tau },y_{\tau };t_{\tau }\right)-d_{\tau }\xi_{000}^{\left(2\right)}-\frac{d_{\tau }\xi_{100}^{\left(2\right)}}{\xi_{200}}\frac{x_{i}-x}{h_{x}}-\\ & & \frac{d_{\tau }\xi_{010}^{\left(2\right)}}{\xi_{020}}\frac{y_{j}-y}{h_{y}}-\frac{d_{\tau }\xi_{001}^{\left(2\right)}}{\xi_{002}}\frac{t_{k}-t}{h_{t}}{]}^{2}K_{ijk}+o_{p}\left(1\right) \end{array}$$

$$\begin{array}{ccc} & = & \frac{1}{NH}\left(\sum_{\left(x_{i},y_{j};t_{k}\right)\in I^{1}}+\sum_{\left(x_{i},y_{j};t_{k}\right)\in I^{2}}\right)\\ & & [f\left(x_{i},y_{j};t_{k}\right)-f_{-}\left(x_{\tau },y_{\tau };t_{\tau }\right)-d_{\tau }\xi_{000}^{\left(2\right)}-\frac{d_{\tau }\xi_{100}^{\left(2\right)}}{\xi_{200}}\frac{x_{i}-x}{h_{x}}-\\ & & \frac{d_{\tau }\xi_{010}^{\left(2\right)}}{\xi_{020}}\frac{y_{j}-y}{h_{y}}-\frac{d_{\tau }\xi_{001}^{\left(2\right)}}{\xi_{002}}\frac{t_{k}-t}{h_{t}}{]}^{2}K_{ijk}+o_{p}\left(1\right)\\ & = & \frac{1}{NH}\sum_{\left(x_{i},y_{j};t_{k}\right)\in I^{1}}[-d_{\tau }\xi_{000}^{\left(2\right)}-\frac{d_{\tau }\xi_{100}^{\left(2\right)}}{\xi_{200}}\frac{x_{i}-x}{h_{x}}-\\ & & \frac{d_{\tau }\xi_{010}^{\left(2\right)}}{\xi_{020}}\frac{y_{j}-y}{h_{y}}-\frac{d_{\tau }\xi_{001}^{\left(2\right)}}{\xi_{002}}\frac{t_{k}-t}{h_{t}}{]}^{2}K_{ijk}+\\ & & \frac{1}{NH}\sum_{\left(x_{i},y_{j};t_{k}\right)\in I^{2}}[d_{\tau }-d_{\tau }\xi_{000}^{\left(2\right)}-\frac{d_{\tau }\xi_{100}^{\left(2\right)}}{\xi_{200}}\frac{x_{i}-x}{h_{x}}-\\ & & \frac{d_{\tau }\xi_{010}^{\left(2\right)}}{\xi_{020}}\frac{y_{j}-y}{h_{y}}-\frac{d_{\tau }\xi_{001}^{\left(2\right)}}{\xi_{002}}\frac{t_{k}-t}{h_{t}}{]}^{2}K_{ijk}+o_{p}\left(1\right)\\ & = & d_{\tau }^{2}\int \int \int_{Q^{\left(1\right)}}{\left[\xi_{000}^{\left(2\right)}+\frac{\xi_{100}^{\left(2\right)}}{\xi_{200}}u+\frac{\xi_{010}^{\left(2\right)}}{\xi_{020}}v+\frac{\xi_{001}^{\left(2\right)}}{\xi_{002}}w\right]}^{2}K\left(u,v\right)K\left(w\right)dudvdw+\\ & & d_{\tau }^{2}\int \int \int_{Q^{\left(2\right)}}{\left[1-\xi_{000}^{\left(2\right)}-\frac{\xi_{100}^{\left(2\right)}}{\xi_{200}}u-\frac{\xi_{010}^{\left(2\right)}}{\xi_{020}}v-\frac{\xi_{001}^{\left(2\right)}}{\xi_{002}}w\right]}^{2}K\left(u,v\right)K\left(w\right)dudvdw\\ & & +o_{p}\left(1\right)\\ & = & d_{\tau }^{2}{\left(C_{\tau }\right)}^{2}+o_{p}\left(1\right). \end{array}$$

### Appendix A.4. Proof of Theorem 3

For the case when $\left(x,y;t\right)\in \Omega_{h}\setminus J_{h}$, the estimator $\hat{f}\left(x,y;t\right)$ is one of $\hat{a}\left(x,y;t\right)$, ${\hat{a}}^{\left(1\right)}\left(x,y;t\right)$, ${\hat{a}}^{\left(2\right)}\left(x,y;t\right)$ and $({\hat{a}}^{\left(1\right)}\left(x,y;t\right)+{\hat{a}}^{\left(2\right)}\left(x,y;t\right))/2$, all of which are consistent estimators of f(x,y;t). So, we have the result in the theorem.

For the case when $\left(x,y;t\right)\in J_{h}\setminus S_{h}$, it is easy to see that we have either i) $e\left(x,y;t\right)=\sigma^{2}+d_{\tau }^{2}{\left(C_{\tau }\right)}^{2}+o_{p}\left(1\right)$, $e^{\left(1\right)}\left(x,y;t\right)=\sigma^{2}+o_{p}\left(1\right)$, and $e^{\left(2\right)}\left(x,y;t\right)=\sigma^{2}+d_{\tau }^{2}{\left(C_{\tau }^{\left(2\right)}\right)}^{2}+o_{p}\left(1\right)$, or ii) $e\left(x,y;t\right)=\sigma^{2}+d_{\tau }^{2}{\left(C_{\tau }\right)}^{2}+o_{p}\left(1\right)$, $e^{\left(1\right)}\left(x,y;t\right)=\sigma^{2}+d_{\tau }^{2}{\left(C_{\tau }^{\left(1\right)}\right)}^{2}+o_{p}\left(1\right)$, and $e^{\left(2\right)}\left(x,y;t\right)=\sigma^{2}+o_{p}\left(1\right)$. In both cases, we have $D\left(x,y;t\right)=d_{\tau }^{2}{\left(C_{\tau }\right)}^{2}+o_{p}\left(1\right)$. Therefore, asymptotically D(x,y;t)>u. Since $e^{\left(1\right)}\left(x,y;t\right)<e^{\left(2\right)}\left(x,y;t\right)$ in i), the estimator $\hat{f}\left(x,y;t\right)$ is ${\hat{a}}^{\left(1\right)}\left(x,y;t\right)$ in this case, which is a consistent estimator of f(x,y;t). A similar result follows in the case ii).
